# Disrupting coupling within mycobacterial F-ATP synthases subunit ε causes dysregulated energy production and cell wall biosynthesis

**DOI:** 10.1038/s41598-019-53107-3

**Published:** 2019-11-14

**Authors:** Wuan-Geok Saw, Mu-Lu Wu, Priya Ragunathan, Goran Biuković, Aik-Meng Lau, Joon Shin, Amaravadhi Harikishore, Chen-Yi Cheung, Kiel Hards, Jickky Palmae Sarathy, Roderick W. Bates, Gregory M. Cook, Thomas Dick, Gerhard Grüber

**Affiliations:** 1 Nanyang Technological University, School of Biological Sciences, 60 Nanyang Drive, Singapore, 637551 Republic of Singapore; 2 Department of Microbiology and Immunology, Yong Loo Lin School of Medicine, National University of Singapore, 14 Medical Drive, Singapore, 117599 Republic of Singapore; 3 Division of Chemistry and Biological Chemistry, School of Physical and Mathematical Sciences, Nanyang Technological University, 21 Nanyang Link, Singapore, 637371 Republic of Singapore; 4 Department of Microbiology and Immunology, School of Biomedical Sciences, University of Otago, Dunedin, 9054 New Zealand; 5 Center for Discovery and Innovation, Hackensack Meridian Health, 340 Kingsland Street Building 102, Nutley, NJ 07110 USA

**Keywords:** Bacteriology, Pathogens

## Abstract

The dynamic interaction of the N- and C-terminal domains of mycobacterial F-ATP synthase subunit ε is proposed to contribute to efficient coupling of H^+^-translocation and ATP synthesis. Here, we investigate crosstalk between both subunit ε domains by introducing chromosomal *atpC* missense mutations in the C-terminal helix 2 of ε predicted to disrupt inter domain and subunit ε-α crosstalk and therefore coupling. The ε mutant εR105A,R111A,R113A,R115A (ε^4A^) showed decreased intracellular ATP, slower growth rates and lower molar growth yields on non-fermentable carbon sources. Cellular respiration and metabolism were all accelerated in the mutant strain indicative of dysregulated oxidative phosphorylation. The ε^4A^ mutant exhibited an altered colony morphology and was hypersusceptible to cell wall-acting antimicrobials suggesting defective cell wall biosynthesis. *In silico* screening identified a novel mycobacterial F-ATP synthase inhibitor disrupting ε’s coupling activity demonstrating the potential to advance this regulation as a new area for mycobacterial F-ATP synthase inhibitor development.

## Introduction

Tuberculosis (TB), caused by the pathogen *Mycobacterium tuberculosis* (*Mtb*), is one of most important infectious diseases worldwide. One third of the world population is latently infected by *Mtb*. During infection, *Mtb* undergoes a wide range of metabolic changes, which correlate with either replicative growth or non-replicative persistence^1^. Such forms of adaptation include the ability of mycobacteria to respire, regenerate reducing equivalents, and generate the biological energy currency adenosine triphosphate (ATP) via oxidative phosphorylation. During the latter, an electrochemical gradient of H^+^ is formed by the electron transport chain (ETC), essential for the survival of replicating and non-replicating mycobacteria, and driving the energy converter, F_1_F_O_ ATP synthase^2,3^. Phenotypic screening identified the antitubercular drug bedaquiline (BDQ, Sirturo), inhibiting the mycobacterial F-ATP synthase^4^. However, the successful advance of this drug has been overshadowed by the development of clinical resistance^5,6^.

**Figure 1:**
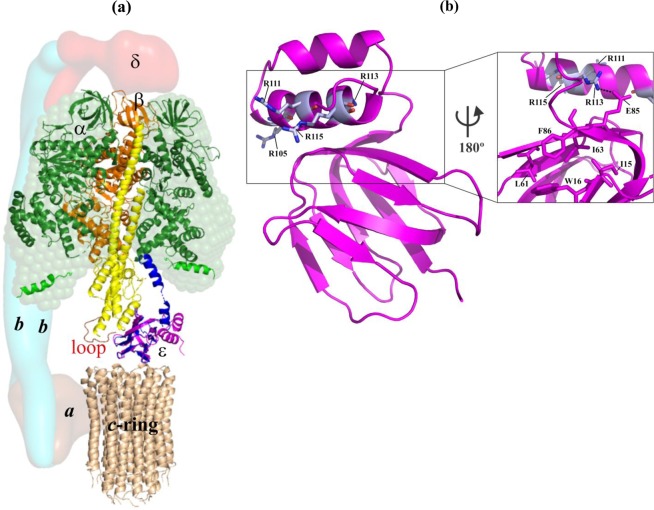
Structural features of the F-ATP synthase and the mycobacterial coupling subunit ε. (**a**) The structural model of the mycobacterial F-ATP synthase was generated based on the cryo-EM model of *E. coli* F-ATP synthase (PDB ID: 5T4O)^47^ and the structural model of the α_3_β_3_γε complex of the mycobacterial F-ATP synthase^7^. Subunits α (*green*), β (*orang*e), γ (yellow), and ε (*magenta*) are from the *M. smegmatis* crystal structure (PDB ID: 6FOC)^7^, whereby the missing β-sheet elements of subunit γ were added by the respective subunit γ elements of the *E. coli* F-ATP synthase (PDB ID: 5T4Q). Mycobacterial subunit γ has a unique γ-loop^13^ which is highlighted in *red*. The solution shape of α^chi^ is displayed as *green sphere*^14^. The *c*-ring loop residues of *M. phlei* (*wheat*; PDB ID: 4V1G^9^), proposed to interact with the rotating ε and γ stalks subunits are indicated. The modelled extended ε subunit (*blue*) of *M. tuberculosis* (PDB ID: 5YIO) reaches the DELSEED-region in subunit α^10^. The low-resolution shapes of the related *E. coli* subunits *a*, *b-dimer* and δ are shown in the *brown*, *light blue* and *red*, respectively, and based on the EM map (PDB ID: 5T4O). (**b**) NMR solution structure of *Mt*ε (PDB ID: 5YIO)^10^ showing R105A, R111A, R113A, and R115A (*magenta*) in helix-2 of the C-terminal domain. (Inset) Closer view of the back surface revealing the interaction of R113 with E85 of the N-terminal domain, which is in close proximity to the hydrophobic cleft proposed to bind BDQ. We thank Dr. S. S. M. Malathy for the art work of (**a**).

The mycobacterial F_1_F_O_ ATP synthase consists of the F_O_ complex (subunits *a*:*b*:*b’*:*c*_9_), responsible for H^+^-transfer from the periplasmic- to the cytoplasmic side. The water soluble F_1_ part (subunits α_3_:β_3_:γ:ε)^7^ uses the H^+^-motive force to synthesize ADP and P_i_ to ATP within the three catalytic αβ-pairs (Fig. 1a). The latter is linked with the F_O_ domain by the two rotating central stalk subunits γ and ε, and the peripheral stalk subunits *b*, *b’* and δ^8^. In the F_O_ part, each of the nine *c* subunit forms a helix-loop-helix structure^9^. The loop docks to the bottom of the N-terminal domain (εNTD) of subunit ε^10^ and the globular domain of γ (Fig. 1a). Both subunits ε and γ rotate and allow the coupling to the F_1_ portion to transfer torque^11^. Interestingly, the mycobacterial F-ATP synthase is unable to establish a significant H^+^-gradient due to ATP hydrolysis. In addition, the enzyme reveals low or latent ATPase activity^12,13^. Recently, specific amino acids in the nucleotide-binding subunit α, and the subunits γ and ε have been shown to be in part responsible for latent ATPase activity and the lack of ATP hydrolysis-driven H^+^-pumping^10,13,14^.

The structure of the rotary *Mtb* ε subunit (*Mt*ε) (PDB ID: 5YIO), composed of 121 residues, consists of an N-terminal β-barrel (residues M1-E87) connected by a two residues linker (S88-E89) to the helix-loop-helix fold of the C-terminal domain (CTD; residues I90-D121) (Fig. 1b)^10^. Compared to other ε subunits^15–18^, the two α-helices of the CTD of *Mt*ε are shorter^10^. Its second helix interacts via its residues A108, R109, A112, and R113 with D47, D48, and A49 of the *Mt*εNTD^10^. Furthermore, amino acids E87-D91 and A112-V117 interact, which stabilizes the so-called compact (ε_c_) conformation of the protein in solution. We recently demonstrated that very C-terminal residue of *Mt*ε can be cross-linked with the C-terminal DELSEED-region of subunit α. These data showed that the *Mt*εCTD can undergo a switch from a compact- (ε_c_), a conformation also displayed in the recent structure of the *M. smegmatis* F_1_-ATPase^7^, to an extended conformation (ε_e_)^10^, that would allow coupling of *c*-ring rotation via a spring-like mechanism inside the subunit ε up to the nucleotide-binding and catalytic subunits. Based on solution NMR data of *Mt*ε as well as genetic and enzymatic studies, a model was proposed in which the neighborhood of *Mt*εD121 and *Mt*εR115 of helix α2 come close to the DELSEED-part of subunit α (Fig. 1a)^10^. Residue R113 in helix α2 is predicted to interact with a second α-subunit via amino acid V117 (Fig. 1a)^10^. These connections of *Mt*ε with subunits α-β would give sufficient interaction to enable ATP synthesis and ATP hydrolysis inside the catalytic α_3_:β_3_-headpiece.

Here, we used a complementary multidisciplinary approach to shed light on the essentiality of crosstalk between the NTD and CTD within mycobacterial subunit ε and the catalytic headpiece for ATP formation by introducing chromosomal missense mutations (R105A, R111A, R113A and R115A) in the C-terminal helix 2 of *M. smegmatis* mc^2^ 155 F-ATP synthase subunit ε (ε(R105A,R111A,R113A,R115A) mutant), called ε^4A^ throughout the text, predicted to interrupt inter-domain and subunit ε-α crosstalk and therefore coupling. The cell morphology of the mutant bacilli changed, and the overall cell length was only one-third of the size of wild-type bacteria (WT). Substitution of these amino acids caused a more than 10-fold drop in ATP synthesis and a moderate reduction in ATP hydrolysis of inverted membrane vesicles (IMVs) derived from *M. smegmatis* ε^4A^ mutant as well as a reduction in the intracellular ATP level of intact bacteria, when grown in minimal media. Both ETC- and carbon metabolism activities were increased in the mutant strain, which is discussed in context of how the TB drug BDQ works. Generating recombinant subunit ε mutants provided structural insights into alterations of the residues necessary for the mechanistic talks between the N- and C-terminal domains of this subunit. Finally, a new mycobacterial F-ATP synthase inhibitor was discovered.

## Results

### Effect of epsilon regulation of F-ATP synthase on bacterial growth kinetics and cellular energetics

**Figure 2:**
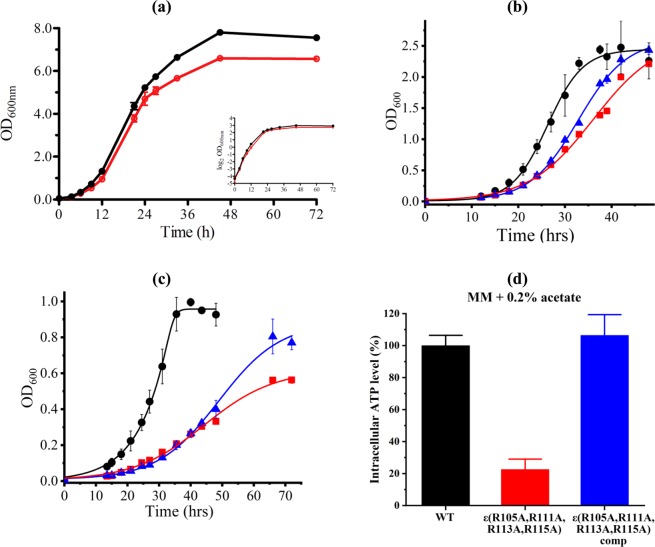
Growth in 7H9 medium and HdB minimal medium. (**a**) *M. smegmatis* WT (*black*) and mutant ε^4A^ (*red*) were grown in 7H9 media containing 0.2% glycerol. (**b,c**) Comparison of growth of *M. smegmatis* WT (*black*), ε^4A^ mutant strain (*red*) and its complemented mutant strain (*blue*) in HdB minimal media containing (**b**) 0.2% glycerol or (**c**) 0.2% acetate. Growth was determined by measuring cell density using optical density at 600 nm (OD_600_). The growth differences of all three strains could be observed in both growing conditions, in which the ε^4A^ mutant strain grew slower than WT strain, and the growth was partially rescued by introducing pMV262-*atpC* into the mutant strain. (**d**) Intracellular ATP levels measured in WT, ε^4A^ mutant and ε^4A^ complemented strain at mid-log phase in minimal media containing 0.2% acetate. All growth experiments are performed in three biological replicates, and the error bar represents standard deviations.

In order to determine whether the arginine residues R105, R111, R113 and R115 with the NTD of mycobacterial subunit ε are essential for the crosstalk between both the NTD and the CTD, the *M. smegmatis* mc^2^155 F-ATP synthase mutant ε^4A^ with the substitution of the four arginine residues inside subunit ε to alanine was engineered. Targeted sequencing confirmed the presence of the desired mutations. Whole genome sequencing performed on both WT and the ε^4A^ mutant suggests that there are only several minor polymorphisms (Table S1). The ε^4A^ mutant and isogenic WT strain were first examined in rich 7H9 nutrient liquid medium supplemented with 0.2% glycerol. The maximum specific growth rate of the ε^4A^ mutant in this medium was similar to that of the WT strain (Fig. 2a). When grown in minimal medium containing 0.2% glucose, the mutant (doubling time of 8.9 h) grew significantly slower than the WT strain (doubling time of 6.4 h), and the final optical density was lower in the ε^4A^ mutant (Fig. S1a,b), which can be seen directly from their respective cell cultures at different time points (Fig. S1b). It is noteworthy that the mutant formed clumps in minimal media (Figs S1b and S2). Furthermore, slower bacterial growth correlated with a reduced intra-bacterial ATP level (Fig. S1c). In addition, when grown in Hartmans-de Bont (HdB)) minimal medium containing 0.2% glycerol (fermentable carbon source), the ε^4A^ mutant grew significantly slower than the WT strain, with doubling times of 4.1 h and 3.2 h, respectively (Fig. 2b). The growth was partially rescued in the ε^4A^ mutant complemented with the *atpC* gene from WT (Fig. 2b). Despite the ε^4A^ mutant growing slower on glycerol, no significant differences were observed between all three strains in terms of their dry weight, molar growth yield on glycerol, final external pH and optical density at 600 nm (OD_600_) (Table 1). Since glycerol is a fermentable carbon source, and the F-ATP synthase is involved in oxidative phosphorylation, the non-fermentable carbon source acetate was used to exclude any energy production contributed from substrate level phosphorylation during bacterial growth. Under this condition, the growth of ε^4A^ mutant was slower and significantly delayed when compared to the WT strain (7.9 h vs 4.4 h, respectively; Fig. 2c). A comparison of the stationary phase (growth at 72 h) for all three strains showed that the complemented strain grew to a final OD_600_ that was close to that of the WT strain, while the ε^4A^ mutant had a ~30% reduction in final OD_600_ (Fig. 2c). In this context, we do not exclude that there are possibilities that phenotypes those were not complemented by the expression of WT (i.e. Fig. 2b,c) were due to the effect of the background mutations.

**Table 1:** Growth kinetics of the *M. smegmatis* WT and ε^4A^ mutant strain and its complemented strain in HdB minimal media supplemented with 0.2% glycerol or acetate.

	WT strain	ε^4A^ mutant strain	ε^4A^ complemented strain
**In HdB media supplemented with 0.2% glycerol**			
Doubling time (h)	3.2	4.1	3.9
Dry weight at 48 h (g/l)	1.07 ± 0.02	0.98 ± 0.04	1.04 ± 0.02
Y_glycerol_ (g/mol) at 48 h	33.07 ± 1.28	33.91 ± 2.96	37.68 ± 1.70
pH at 48 h	5.84 ± 0.04	5.95 ± 0.02	5.95 ± 0.03
OD_600_ at 48 h	2.26 ± 0.29	2.21 ± 0.03	2.43 ± 0.07
**In HdB media supplemented with 0.2% acetate**			
Doubling time (h)	4.4	7.9	7.6
Dry weight at 72 h (g/l)	0.38 ± 0.01	0.24 ± 0.01	0.29 ± 0.02
Y_acetate_ (g/mol) at 72 h	9.45 ± 0.78	5.78 ± 0.47	7.47 ± 0.43
pH at 72 h	8.30 ± 0.03	7.86 ± 0.05	8.02 ± 0.10
OD_600_ at 72 h	0.79 ± 0.04	0.56 ± 0.02	0.77 ± 0.04

Reduced growth correlated with a dramatically lower intracellular ATP level in the ε^4A^ mutant when compared to the WT strain (Fig. 2d). The ATP level of the complemented strain was restored to WT values (Fig. 2d). The molar growth yield on acetate [Y_acetate_; dry weight of cells (g) produced with one mol of acetate utilized] was 5.78 ± 0.47 g/mol in the ε^4A^ mutant compared to 9.45 ± 0.78 g/mol for the WT strain (Table 1). The external pH of the ε^4A^ mutant culture on acetate was more acidic (despite less biomass) compared to the WT strain (Table 1). These data demonstrate that carbon sources like acetate that are strictly coupled to the F-ATP synthase are metabolized less efficiently in the ε^4A^ mutant compared to the WT strain.

**Figure 3:**
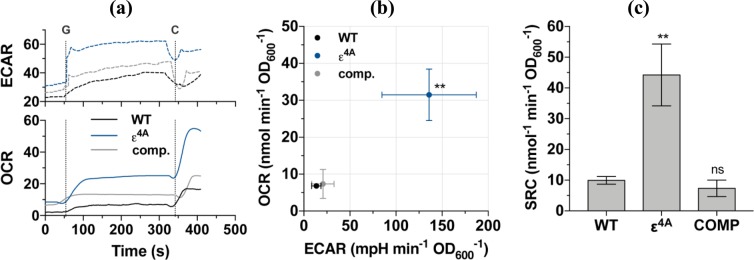
Oxygen consumption and extracellular flux analysis of mutant ε^4A^. (**a**) Raw trace schematic of experimental setup. Respiration was initiated with 0.2% glucose and subsequently uncoupled with 10 µM CCCP at points “G” and “C”, respectively. (**b**) Baseline-subtracted OCR *vs* ECAR phenograms of *M. smegmatis* strains after glucose-stimulated respiration. (**c**) The spare respiratory capacity (SRC, calculated as [OCR_CCCP_ - OCR_glucose_]) of *M. smegmatis* strains.

The growth rate and molar growth yield of the ε^4A^ mutant during growth on acetate was significantly lower than the WT strain suggesting that regulation by the epsilon subunit was crucial for efficient energy coupling. To assess the bioenergetic properties of the ε^4A^ mutant compared to the isogenic WT parent and complemented strain in more depth, we performed extracellular flux analysis (Fig. 3a–c). In this assay, oxygen consumption rate (OCR) represents the activity of the respiratory chain, while the extracellular acidification rate (ECAR) represents the activity of carbon metabolism and the TCA cycle^19^. Both respiration and carbon metabolism activity were significantly increased in the ε^4A^ mutant: the OCR of the mutant was 4.6-fold higher and the ECAR was 10.1-fold higher than WT cells (Fig. 3a). This profile would be indicative of an uncoupled cell with a depolarized membrane, as the uncoupler carbonyl cyanide *m*-chlorophenyl hydrazine (CCCP) causes an increase in both OCR and ECAR^19^. If true, CCCP should not stimulate OCR in the ε^4A^ mutant. However, the spare respiratory capacity (the degree of OCR stimulation by canonical uncouplers like CCCP) was increased by 4.5-fold in the ε^4A^ mutant (Fig. 3c). Taken together, these data suggest that the ε^4A^ mutant has upregulated respiratory and carbon metabolism pathways in order to maintain a fully energized membrane. We propose that the substitution of the four C-terminal arginines in the ε subunit leads to either increased uncoupled H^+^-pumping- and/or ATP synthesis activity of the F-ATP synthase, or dysregulated entry of protons into the cytoplasm: both of which require increased respiration to compensate (see below).

### BDQ susceptibility is enhanced in the ε^4A^ mutant strain

Recently it has been shown that C-terminal residues of *Mt*ε, including R109 and R115, undergo changes in the chemical shift upon binding of BDQ to the NTD of subunit ε, which appears to be due to interactions between the NTD and CTD^10^. To determine whether the amino acid replacement inside the ε^4A^ mutant alters BDQ susceptibility of the bacterium, growth inhibition dose-response curves were determined using the broth dilution method as described earlier^20^. As shown in Fig. S3a, the mutation of R105, R111, R113 and R115 to alanines render the mutant strain more sensitive to BDQ with a 3-fold shift in minimal inhibitory concentration (MIC_50_) from 10.3 nM to 3.2 nM. Since BDQ is known to have delayed bactericidal activity during the first 3–4 days^20^, we measured the effect of BDQ on cell viability (Fig. S3b–e). As displayed in Fig. S3b, BDQ showed very little bactericidal activity against WT *M. smegmatis* mc^2^ 155 during the first two days of culture, reflected by an increasing cell number of about 1.1 log_10_ units in CFUs. Such increase in cell viability over the first two days was repeatedly not observed in the ε^4A^ mutant (Fig. S1c), indicating that the altered BDQ susceptibility went in part along with bacterial killing, and that subunit ε plays a critical role in it.

### Effect of the ε^4A^ mutation on bacterial morphology and resistance to cell wall antimicrobials

**Figure 4:**
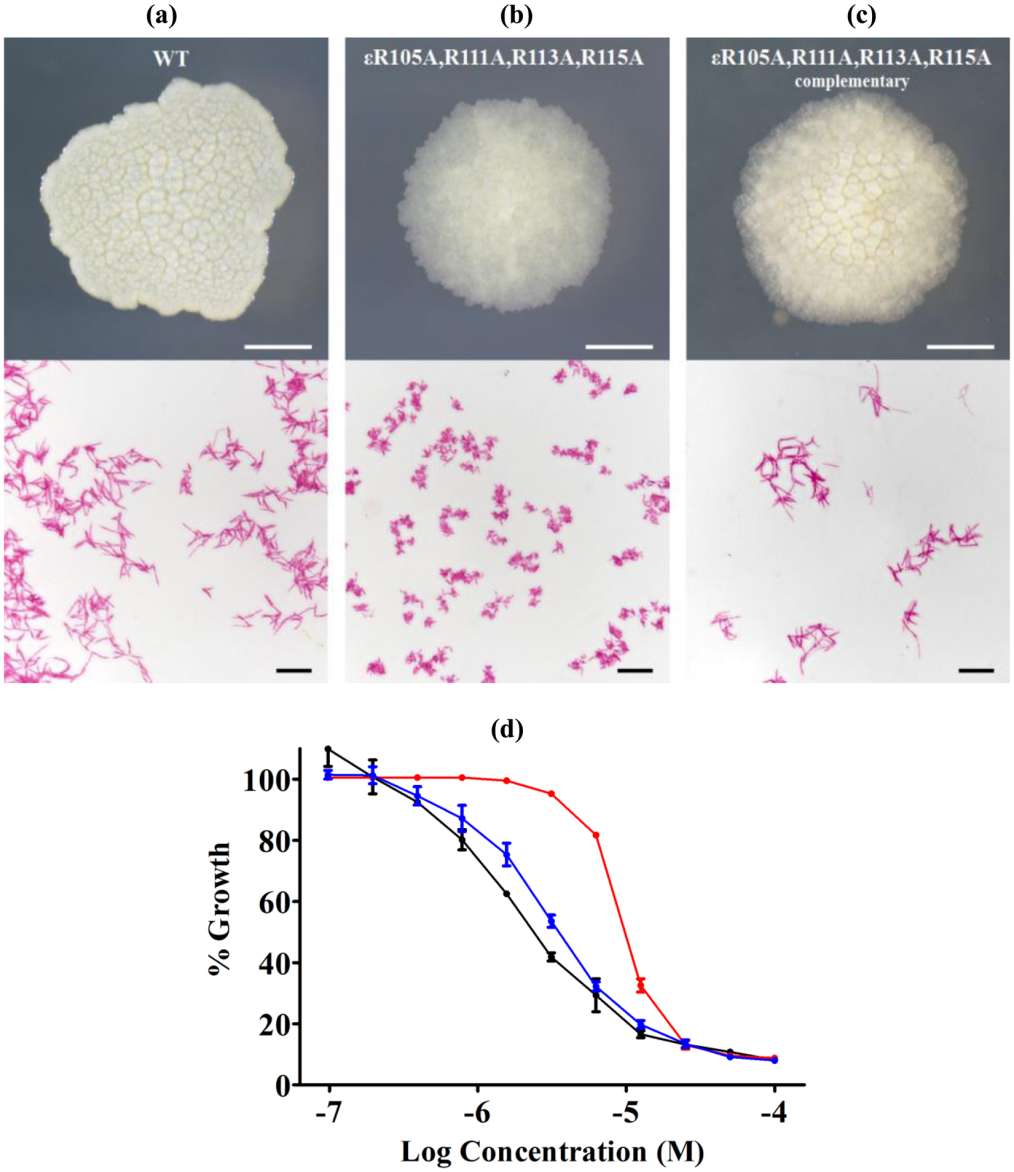
Growth and cell size phenotype of *M. smegmatis*. (**a–c**) (Top) The bacterial colony morphology on solid medium and (Bottom) acid-fast stained log phase culture samples of *M. smegmatis* (**a**) WT, (**b**) ε^4A^ mutant and the (**c**) ε^4A^ complemented mutant. Colonies are shown after four days of incubation. (**d**) Meropenem growth inhibition dose-response curves of *M. smegmatis* WT (*black*), ε^4A^ mutant (*red*) and the ε^4A^ complemented strain (*blue*). The experiments were performed in triplicate. Data are shown with their standard deviations.

Because of the growth differences between the ε^4A^ mutant and WT, the effect of the introduced mutation was further investigated in terms of colony size and cell morphology (Fig. 4). The WT *M. smegmatis* strain and the ε^4A^ mutant were plated on 7H10 agar plates and similar colony sizes were observed for both the WT and the mutant strain (Fig. 4a–c). Interestingly, the irregular opaque colonies of WT with their flat surface were replaced by a circular translucent granular feature with a slightly elevated center in the ε^4A^ mutant (Fig. 4a,b). This phenotype was partially restored when ε^4A^ mutant was complemented with the WT *atpC* gene (Fig. 4c). Surprisingly, when observed under a light microscope, the ε^4A^ mutant bacilli displayed a dramatically shorter cell length of 1.63 ± 0.38 μm, which is only one-third of the size of WT (4.60 ± 1.26 μm) (Fig. 4b). Due to the difference in colony morphology and cell size in ε^4A^ mutant, further investigation was done on the susceptibility of WT *M. smegmatis*, ε^4A^ mutant and ε^4A^ complemented mutant strains to the β-lactam antibiotic meropenem that targets cell wall synthesis of the bacteria^21^. Growth inhibition dose-response curves were determined using the broth dilution method as described earlier^21^. As shown in Fig. 4d, the ε^4A^ mutant revealed a reduced susceptibility by meropenem resulting in a minimum inhibitory concentration (MIC_50_) of 10 μM. In comparison, WT and the complemented strain showed a similar inhibitory curve with a MIC_50_ of 3 μM. The effect of meropenem on the cell viability of these mutants was determined (Fig. S4). While meropenem at a concentration of 3x MIC_50_ did not affect cell viability against WT during the first four days of incubation (Fig. S4a,c), the same inhibitor concentration reduced moderately and reproducibly cell viability of the ε^4A^ mutant (Fig. S4b,d). In comparison, at 30x MIC_50_ a drop of about 1.4 log_10_ units and 2.8 log_10_ units in CFUs was observed for the WT and ε^4A^ mutant strain, respectively (Fig. S4a,b).

### The crosstalk between the C-terminal helix and the NTD of subunit ε is critical for ATP synthesis and -hydrolysis

**Figure 5:**
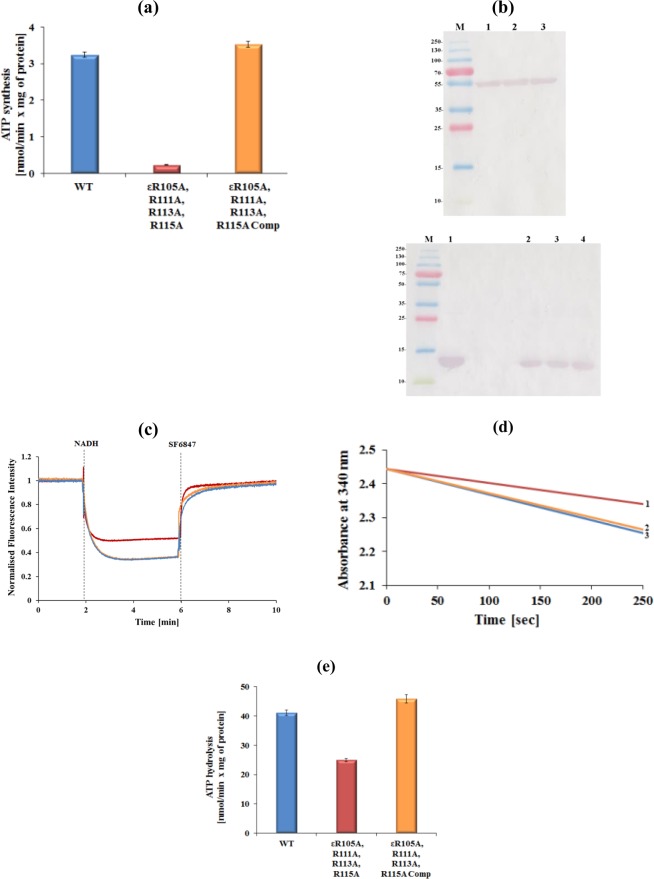
Catalytic activities of *M. smegmatis* F-ATP synthase WT and mutant proteins. (**a**) ATP synthesis measured for WT (*blue*), ε^4A^ mutant (*red*) and ε^4A^ complemented mutant (*orange*) IMVs of *M. smegmatis*. (**b**) (*above*) Western blot of an SDS-gel showing similar content for WT mycobacterial F-ATP synthase (1) as well as for the ε^4A^ mutants (2) and for ε^4A^ complemented mutant (3) as detected by an antibody against the catalytic β subunit of the *Escherichia coli* F-ATP synthase. The Western blot below reveals in lane 1 recombinant *Mt*ε; lane 2 WT IMVs; lane 3 ε^4A^ mutant IMVs and lane 4 ε^4A^ complemented mutant IMVs. Antibodies against subunit ε of the *E. coli* F-ATP synthase were used for the analysis. (**c**) Fluorescence quenching of ACMA by IMVs of the WT (*light blue*), ε^4A^ mutant (*red*), and its complemented mutant (*orange*) after addition of 2 mM NADH. The uncoupler SF6847 was added at the indicated time point to collapse the H^+^-gradient. The experiments were performed in triplicate. (**d**) Continuous ATPase activity of WT (*blue*) *M. smegmatis* F-ATP synthase, ε^4A^ (*red*) and ε^4A^ complemented mutant (*orange*), respectively, using IMVs measured in the presence of NDH-2 inhibitor thioridazine (80 μM) and 2 mM MgATP. (**e**) Specific ATPase activity of WT, ε^4A^ mutant and ε^4A^ complemented mutant IMVs. Values are the mean of six determinations with two different IMV batches of WT and mutants.

To confirm that the reduction of intracellular ATP formation is related to the effect of the C-terminal substitutions of the ε^4A^ mutant, ATP synthesis was investigated using IMVs. As demonstrated in Fig. 5a, the IMVs of WT revealed an ATP synthesis activity of 3.24 ± 0.07 nmol min^−1^ (mg total protein)^−1^. In contrast, when IMVs containing the F-ATP synthase mutant ε^4A^ were used, low ATP synthesis of only 0.23 ± 0.01 nmol min^−1^ (mg total protein)^−1^ was observed. To confirm that the substitution of the four C-terminal arginine residues of subunit ε causes the reduction of ATP synthesis activity, the IMVs of the complemented mutant was studied, revealing an ATP synthesis activity of 3.53 ± 0.08 nmol min^−1^ (mg total protein)^−1^, which is similar to WT IMVs (Fig. 5a). Since the Western-blot analysis in Fig. 5b confirms that the amount of F-ATP synthases located in the ε^4A^ mutant vesicles was comparable with that of the WT and complemented mutant vesicles, and that subunit ε is present within the enzyme complex, the data demonstrate the crucial role of the mycobacterial subunit ε residues R105, R111, R113 and R115 in ATP synthesis.

Since we observed increased OCR and ECAR in the ε^4A^ mutant but reduced ATP synthesis of the ε^4A^ mutant IMVs, we tested whether ε^4A^ mutant IMVs allow H^+^ to move freely across the membranes or whether the increased H^+^-pumping activity is uncoupled to ATP synthesis. Therefore, we firstly tested the leakiness of WT, ε^4A^ mutant and ε^4A^ complemented mutant IMVs to protons in the presence of the fluorescent dye 9-amino-6-chloro-2-methoxyacridine (ACMA). As shown in Fig. 5c, in the presence of ADP + P_i_, but absence of NADH and the uncoupler SF6847, neither the WT, nor the ε^4A^ mutant or ε^4A^ complemented IMVs showed a change in fluorescence indicating that the IMVs were intact, which was confirmed by the significant and fast quenching of ACMA after addition of NADH as well as the increase in fluorescence observed upon addition of SF6847. Interestingly, in the presence of NADH, WT- and ε^4A^ complemented mutant IMVs revealed a more drastic fluorescence quenching compared to the ε^4A^ mutant IMVs, reflecting an increased H^+^-conduction of the F_O_ domain within the ε^4A^ mutant to the outside without coupled ATP synthesis.

In parallel, the effect of the ε^4A^ mutant on ATP hydrolysis was investigated using IMVs. As demonstrated in Fig. 5d,e, IMVs of WT *M. smegmatis* revealed an ATPase activity of about 41.15 ± 0.99 nmol min^−1^ (mg total protein)^−1^, underlining recent results, which demonstrated that *M. smegmatis* hydrolyses ATP, albeit at a relatively low level^12,13^. The IMVs of the ε^4A^ mutant showed an ATPase activity of 24.9 ± 0.5 nmol min^−1^ (mg total protein)^−1^. Thus, the mutant caused a 40% decrease in ATPase activity, when compared to the ATPase activity of the WT enzyme (Fig. 5e). Similar to WT IMVs, the complemented mutant revealed an ATP hydrolysis activity of 45.9 ± 1.3 nmol min^−1^ (mg total protein)^−1^), demonstrating that the mutation of the four C-terminal arginines to alanine causes the alteration of ATPase activity.

### Alterations of a monomeric to an oligomeric form of *Mt*ε due to arginine substitutions

**Figure 6:**
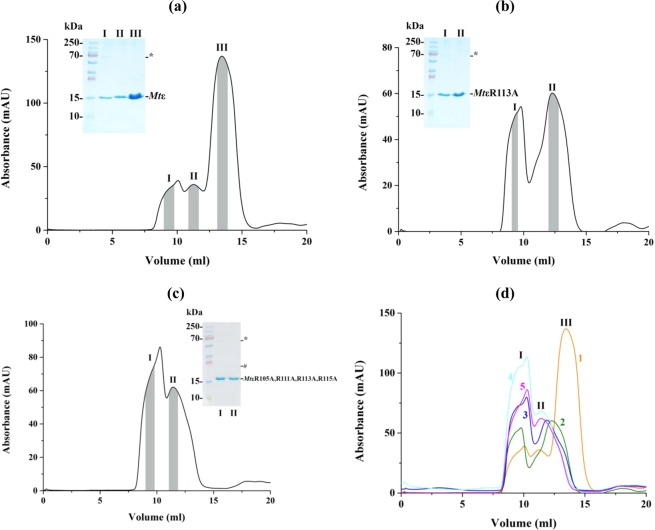
Recombinant protein purification of *Mt*ε and its mutants. (**a**) Three peaks were detected at 9.5, 11.5 and 13.8 ml in the SEC elution profile of *Mt*ε. A higher oligomer band (*; around 70 kDa) and a monomer at 15 kDa were observed in the SDS gel in the inset. (**b**) Elution profile of *Mt*R113A revealed two peaks at 9.5 and 12.5 ml. Grey area under each peak outlines the range of eluates collected and applied on lanes I and II of the respective gel. (**c**) In comparison, the elution diagram of the mutant *Mt*εR105A,R111A,R113A,R115A showed a major peak at 9.5 ml and a second peak which eluted earlier at 11.5 ml. The respective peak samples were applied onto the lanes I and II of the SDS-gel presented as an inset. * and # indicate a higher- and lower oligomer. (**d**) Overlay of SEC chromatograms for WT *Mt*ε and the mutants, where (1) WT, (2) *Mt*εR113A, (3) *Mt*εR113A,R115A, (4) *Mt*εR111A,R113A,R115A, and (5) *Mt*εR105A,R111A,R113A,R115A. Each of the three peaks is associated with one of the following states: higher oligomer (I), lower oligomer (II), and monomeric (III).

To understand the effects of the ε mutant described above in more detail, the four *Mt*ε mutants *Mt*εR113A, *Mt*εR111A,R113A, *Mt*εR111A,R113A,R115A, and *Mt*εR105A, *Mt*εR111A,R113A,R115A were genetically engineered. The overall protein production and solubility of the four mutants were similar to recombinant WT *Mt*ε. WT *Mt*ε and its four mutant proteins were isolated via Ni-NTA affinity purification, followed by a size-exclusion (SEC) step. The SEC chromatogram of WT *Mt*ε showed the peaks I and II at 9.5 ml and 11.5 ml, which correspond to two different oligomeric formations of *Mt*ε, and the major peak III (13.8 ml), representing monomeric *Mt*ε (Fig. 6a)^10^. In comparison, the single mutant *Mt*εR113A revealed the higher oligomer peak I (9.5 ml) and a peak II at 12.5 ml with similar absorbance (Fig. 6b), indicating an additional lower oligomeric form of the protein and that the R113 to alanine substitution shift the equilibrium from a monomer to oligomeric forms. Interestingly, the elution profile of the double mutant *Mt*εR111A,R113A showed that peak II shifted to even a smaller elution volume compared to peak II of mutant *Mt*εR113A and therefore an increase of the oligomeric form (Fig. S5a). In case of the triple and quadruple mutants the ratio of peak I to peak II at 9.5 and 11.5 ml increased (Figs 6c, S5b), reflecting that the additional R to A substitution increase higher oligomer formation. The shifts in elution volume due to the mutations engineered are highlighted by the overlay of all five elution diagrams (Fig. 6d). These data demonstrated that substitution of R113 led to a shift of monomeric to oligomeric *Mt*ε, which increased by the additional substitution of R115 to alanine. Finally, the formation of *Mt*εR111A,R113A,R115A, and *Mt*εR105A,R111A, R113A,R115A prevents the formation of any monomeric *Mt*ε.

### Structural changes of *Mt*εR113A and *Mt*εR105A,R111A,R113A,R115A

**Figure 7:**
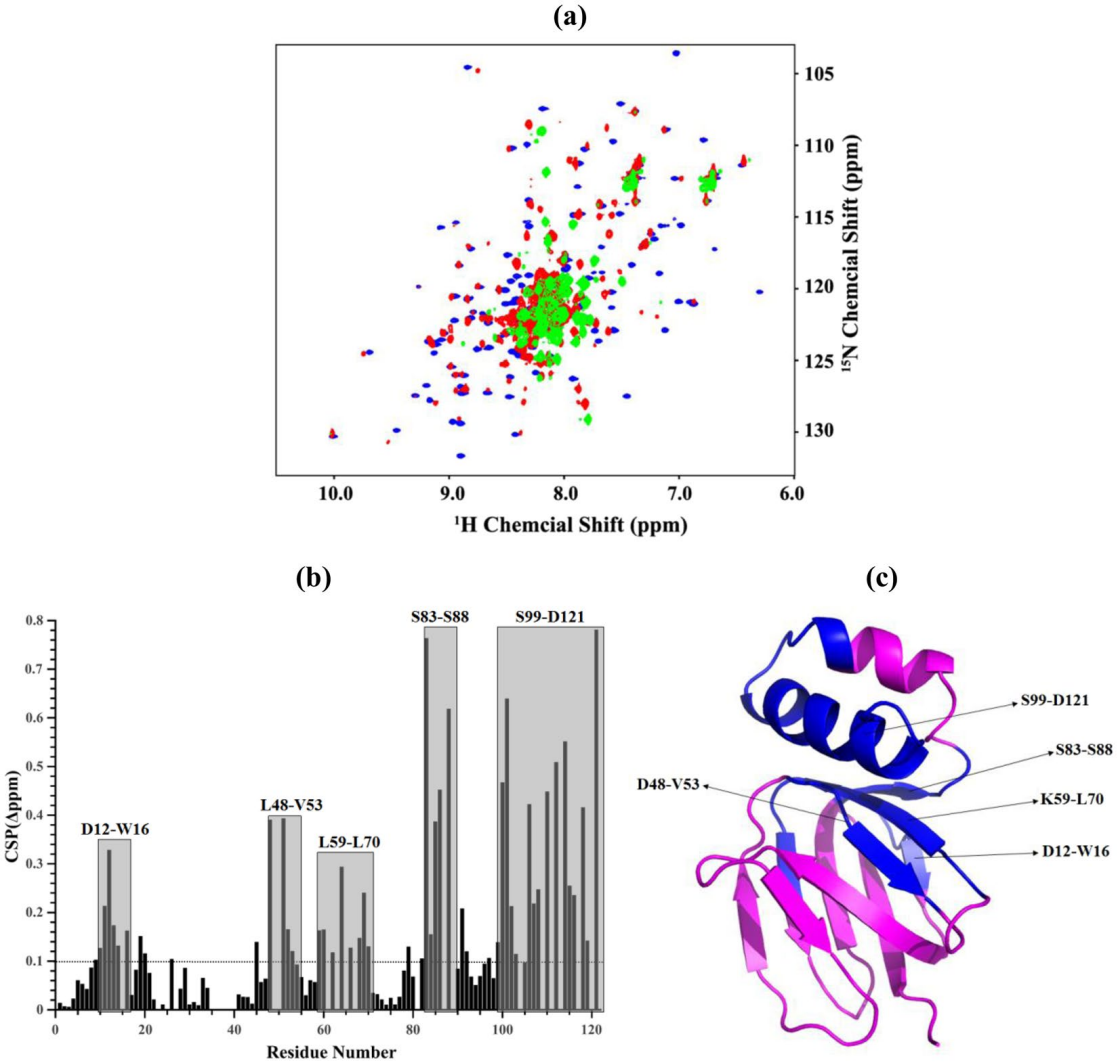
NMR studies of *Mt*ε and its mutants. (**a**) Comparison of overlaid HSQC spectra for *Mt*ε (*blue*), *Mt*εR113A (*red*), and *Mt*εR105A,R111A,R113A,R115A (*green*). (**b**) Weighted Chemical Shift Perturbations (CSPs) for the ^15^N and ^1^H resonance between *Mtε* and *Mt*εR113A; regions with residues showing CSP above the average weighted CSP value are highlighted with *grey* colour and labelled as one-letter code. The weighted CSPs between *Mtε* and *Mt*εR113A for backbone ^15^N and ^1^HN were calculated by the formula Δδ = [(ΔN/5)^2^+(ΔHN)^2^]^0.5^. The average CSP weighted value was displayed by dashed line. (**c**) Ribbon representation of *Mtε* showing the regions and residues with significantly changed chemical shift perturbation values above average (*blue*).

To understand whether structural changes occurred within the single mutant *Mt*εR113A, which eluted in part as a monomer, and the quadruple mutant *Mt*εR105A,R111A, R113A,R115A, NMR experiments were performed with both mutants and the WT protein (Fig. 7). As shown by the overlay of the 2D ^1^H-^15^N HSQC spectra of recombinant *Mt*ε and the mutants, overall structural changes and the presence of a mixed oligomeric- (*Mt*εR113A) or formation of a highly ordered oligomeric state (*Mt*εR105A,R111A,R113A,R115A) were observed (Fig. 7a). Based on the assigned resonance information of WT *Mt*ε, the residues of *Mt*εR113A with significant change in chemical shift were identified and mapped (Fig. 7b,c). The residues D12-W16, L59-L70, the NTD-CTD interacting amino acids L48-V53, and the connecting region between the NTD and CTD (residues S83-S88) were strongly affected by the R113A mutation. In addition, the high chemical shift perturbations (CSPs) values of amino acids within the second helix of the CTD, reflect that the mutation might cause an overall structural rearrangement of the CTD of *Mt*ε (Fig. 7b). The mixed or highly ordered oligomeric states of *Mt*εR113A and *Mt*εR105A,R111A,R113A,R115A indicate that the arginine residues of *Mt*ε are strongly related to the structural stability of the *Mt*ε compact form.

### Identification of a novel mycobacterial F-ATP synthase inhibitor

**Figure 8:**
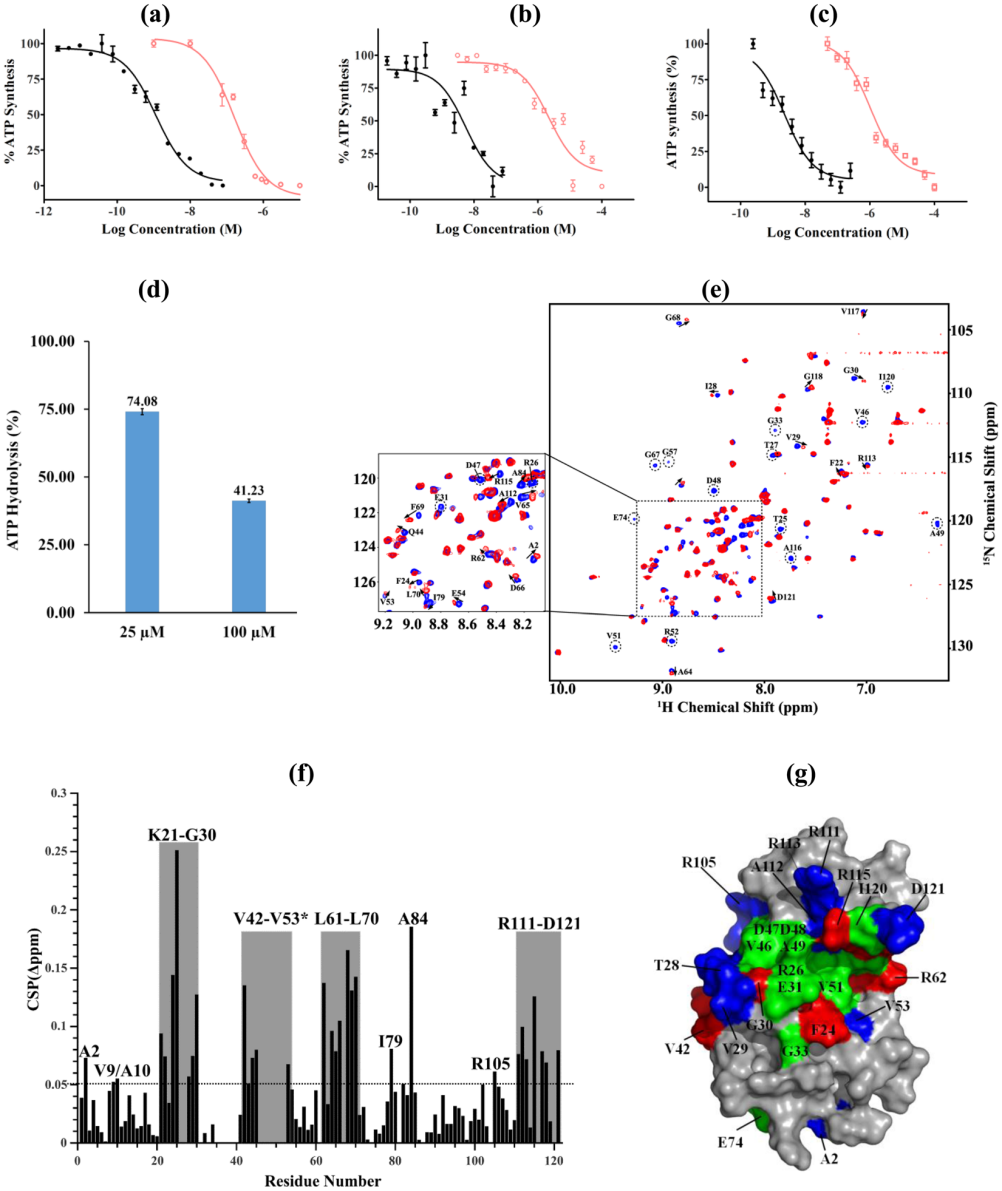
Enzymatic inhibition studies and insights from NMR titrations and docking studies on EGCG binding to *Mt*ε. (**a,b**) Effect of EGCG (○) on NADH-driven ATP synthesis in (**a**) *M. smegmatis* or (**b**) *M. bovis* BCG IMVs. The inhibition profile of BDQ (●) is shown as a control. (**c**) Inhibition of succinate-driven ATP synthesis in *M. bovis* BCG IMVs by EGCG (□) and BDQ (●). (**d**) ATP hydrolysis inhibition of EGCG of the *M. smegmatis* F-ATP synthase of IMVs. (**e**) Selective sections of chemical shift changes of *Mt*ε upon EGCG binding. The changes of chemical shift from compound free *Mt*ε (*blue*) to *Mt*ε-EGCG complex (*red*) are displayed by an arrow. (**f**) Weighted CSPs for the ^15^N and ^1^H resonance of *Mt*ε in the presence of EGCG. CSPs residues showing CSPs above average value (>0.05 ppm) are labelled as one-letter code. The weighted CSPs between *Mt*ε and EGCG for backbone ^15^N and ^1^HN were calculated by the formula Δδ = [(ΔN/5)^2^ + (ΔHN)^2^]^0.5^. (**g**) Mapping of the EGCG-binding surface and conformational changes on *Mt*ε by CSP results; the residues of *Mt*ε revealing more than 0.1 ppm are represented in *red*, while those showing CSP between 0.05 and 0.1 ppm are revealed in *blue*. Disappeared resonances after addition of EGCG are represented in *green*.

Because of the effects of the ε^4A^ on growth, morphology, ATP synthesis, ATP hydrolysis, structural alteration, as well as the recently described dynamics of the NTD and CTD within the *Mt*ε solution NMR structure^10^, and the importance of the mycobacterial ε subunit in coupling^10,12,22^, a model was developed using the *Mt*ε solution structure (PDB ID: 5YIO)^10^ for *in silico* compound screening to identify a novel mycobacterial F-ATP synthase inhibitor (Fig. S6). Together with property filters, molecular docking scores and visual inspection of ligand interaction with the intended arginine residues, 19 compounds were prioritized for experimental characterization. The identified compound was epigallocatechin gallate (EGCG), which is one of the principle polyphenolic compounds found in the leaves of *Camellia sinensis* (tea)^23^. EGCG blocked NADH-driven ATP synthesis of IMVs of WT *M. smegmatis* with a half-maximal inhibitory concentration (IC_50_) of 155.6 ± 1.2 nM (Fig. 8a) and was also a potent ATP synthesis inhibitor of the IMVs of the slow growing *M. bovis* bacillus Calmette–Guérin (IC_50_ = 2.2 ± 0.3 µM; Fig. 8b). In comparison, BDQ was active with an IC_50_ of 1.14 ± 0.2 nM (*M. smegmatis* IMVs) and 7.05 ± 1.32 nM (*M. bovis* BCG). Similarly, EGCG inhibited ATP synthesis of *M. smegmatis* IMVs in the presence of succinate (Fig. 8c), indicating that EGCG does not bind to the NADH-dehydrogenase. To look further into the EGCG-target enzyme, ATP synthesis of IMVs of the BDQ-resistant mutant I66M with the substitution in the *M. smegmatis* subunit *c* was used^24^. As revealed in Fig. S7a, the BDQ-resistant mutant I66M showed a 10-fold reduction in ATP synthesis inhibition by BDQ. Interestingly, the IC_50_ of EGCG with IMVs of the I66M mutant was similar to WT IMVs. Even more, addition of 750 nM of EGCG reduced ATP synthesis of the I66M mutant IMVs significantly (Fig. S7b), confirming that the mycobacterial F-ATP synthase is the enzyme target and that the novel F-ATP synthase inhibitor EGCG shows no cross resistance to BDQ. Furthermore, EGCG also inhibited ATP hydrolysis activity by more than 50% at concentrations of 100 µM in a manner comparable to the known ATPase inhibitors quercetin or BDQ^13^ (Fig. 8d). No uncoupling effect was observed on the WT IMV in the presence of 200 nM of EGCG (Fig. S7c). A clogP value of 1.49 was calculated for EGCG.

### Mechanistic insights into EGCG-binding to *Mt*ε

To confirm binding of EGCG to *Mt*ε and to shed light into the mechanism of action, we carried out NMR titration using the highly resolved and dispersed NMR-spectrum of *Mt*ε (Fig. 8e). The titration of ^15^N-labeled *Mt*ε with EGCG was done in a molar ratio of 1:2 (Fig. 8e). Disappearance of some of the cross-peak intensities in the ^1^H-^15^N HSQC spectrum, major and slight changes of ^15^N- and HN-resonances for some residues, and gradual line broadening with increasing molar ratios were observed, which demonstrate binding of EGCG with *Mt*ε. A plot of CSPs after addition of EGCG to labeled *Mt*ε at a molar ratio of 1:2 are shown in Fig. 8f. Significant changes in chemical shift higher than average plus standard deviation (CSP > 0.1 ppm) were detected for the amino acids F24, T25, G30, V42, R62, D66, G68, F69, A84 and R115. Smaller CSPs over than average (0.05 ppm < CSP < 0.1 ppm) were identified for residues A2, V9, A10, K21, F22, T28, V29, A43-L45, V53, A64, V65 I79, E82, R105, R111-R113, V117, G118 and D121. Residues R26, T27, E31, G33, V46-R52, L61, G67, A116 and I120 showed disappearance of resonances. Most of the amino acid residues affected by EGCG binding are located at the K21-G30, V42-V53, L61-L70 and R111-D121 regions. When mapped onto the NMR solution structure of *Mt*ε (Fig. 8g), most of the residues affected by EGCG have been proposed to be involved in the interaction of the *Mt*εNTD with its second helix and/or in stabilizing the compact (ε_c_) conformation of *Mt*ε in solution^10^.

To corroborate our initial docking result, we have exploited the NMR HSQC data constraints to obtain bound conformation of EGCG-*Mt*ε via molecular docking experiments and evaluated the stability of protein-ligand interactions over 200 nano-seconds by molecular dynamic (MD) studies. Our MD results suggest that EGCG maintains key hydrogen bonding contacts to amino acids D48, D60, and V65 over almost the entire time length of 200 nanoseconds (Fig. S8a). Ligand binding was further stabilized by hydrophobic interactions at A64, I90 and C-terminal residues such as A112 and A116 (Fig. S8b,c). Further, the 5-OH and 5′-OH groups on 2-phenyl and gallic ester were involved in solvent-mediated hydrogen bonding interaction with residues R52, D66 and R62.

## Discussion

### Metabolic adaptation and morphological plasticity of mutant ε^4A^

Mycobacteria are obligate aerobes. They can also live under hypoxic and even anaerobic conditions in a non-replicating state^25,26^. It is proposed that they adapt to a slow growth rate by using a variety of different enzymes like the alternative dehydrogenases and hydrogenases, which keep the flow of reducing equivalents to the ETC when energy is limited. Such adaptation processes can go along with morphological changes as described for the reduction (7-fold) of the *M. smegmatis* cell length in a carbon-limited model^25,26^, reflecting that mycobacteria are able to generate small replicating cells. Here the *M. smegmatis* ε^4A^ mutant caused a significant change in colony morphology, a 3-fold reduction of the cell size, and decreased susceptibility of the antibiotic meropenem. The clump-formation in minimal media may reflect changes in the cell envelope and bacterial surface properties of the mutant strain, which may be related to the reduction in the energy currency ATP, which is important for bacterial cell envelope formation and optimal growth^3,27^.

Interestingly, in 7H9 nutrient media the *M. smegmatis* ε^4A^ mutant showed reduction in biomass compared to WT, reflecting the effect of the mutation on overall growth. Considering the high amount of energy needed to build the cell wall, the morphological plasticity shown by the reduction (3-fold) of the *M. smegmatis* mutant cell size may represent a bacterial strategy to keep producing large cell numbers in an energy limited situation. The lower amount of ATP generated by oxidative phosphorylation may in part be compensated by a higher turnover of the glycolysis pathway and the TCA cycle, in which substrate level phosphorylation provides ATP, and where the reducing equivalents NADH and FADH_2_ will be generated for the subsequent use in the respiratory chain for ATP-formation. In line with this interpretation is the reduced growth of the mutant strain in minimal media with the non-fermentable carbon source acetate. This result is in line with the drop of intracellular ATP of the ε^4A^ mutant, which reached close to WT-level in the complemented strain. Since the TCA cycle has a major biosynthetic role in generating important intermediates for fatty acid-, steroid synthesis as well as amino acid, purines and pyrimidines precursors, a higher TCA cycle turnover would enable sufficient synthesis for the synthesis of proteins, DNA, and the cell wall. In addition, malate, formed within the TCA cycle, can be used to form pyruvate and further refill the pool of glycolytic intermediates by gluconeogenesis^28^. Such processes have been shown to be essential in TB infection in mice and dormancy^29^. Furthermore, the observed reduction of the ε^4A^ mutant in external pH under acetate conditions may alter expression of genes encoding enzyme of the glyoxylate pathway and/or TCA cycle as described for the isocitrate lyase of *M. tuberculosis*^30^. The enzyme provides a short-cut of the TCA cycle resulting in glyoxylate and succinate^28^, with the latter providing the electrons for oxygen reduction within the ETC, which in part may reflect the increased oxygen consumption rate observed for the ε^4A^ mutant. Such remodelling has been demonstrated for *M. tuberculosis* in adaptation to hypoxia^31^.

Similar to the activated respiration and metabolic rerouting within the ε^4A^ mutant, BDQ has been shown to activate the respiration of *M. tuberculosis* and *M. smegmatis*^5,19,32^ leading to dysregulation of mycobacterial metabolism^19^. Recent work has shown that this stimulation of respiration is due to the binding of BDQ to the F-ATP synthase enabling the drug to form an uncoupled microenvironment, by antiporting H^+^/K^+^. This finally, leads to the stimulation of respiration via an ionophoric-like mechanism^33^. We propose that disrupting the ε-subunit function may lead to localized uncoupling in the F-ATP synthase and activation of respiration to compensate. This futile cycle of H^+^/K^+^ exchange would lead to de-energization of the cells through a loss of cellular ATP and the H^+^-motive force ultimately leading to cell death.

An adaptation of the small replicating *M. smegmatis* ε^4A^ cells to energy limitation is also indicated by its longer doubling time compared to the WT strain minimal media containing 0.2% acetate. DNA replication is regulated through several mechanisms, including the ratio of ATP:ADP and the level of the initiator protein DnaA^34^, which unwinds the chromosomal origin (*oriC*) and complexes with ATP. The DNA-dependent unwinding of *oriC* needs ATP in a millimolar range (*K*_*d*_ ~ 1 mM). Therefore, the concentration of available ATP is important, which correlates with the total amount of intracellular ATP. At this point of time we can only speculate, whether the low concentration of ATP within the mutant strain and cell-cycle dependent fluctuations in the energy currency levels may affect initiation frequency and finally the doubling time.

### Mechanistic and structural influence of the subunit ε mutations

The recent *Mt*ε structure showed connections between the amino acids A10-W16 with the regions L61-A64 and A81-I90, respectively^10^. Based on these as well as the atomic structure of the mycobacterial *c*-ring^9^ and ^15^N relaxation data of *Mt*ε^10^, a structural and mechanistic model was described^10^, in which rotation of the *c*-ring would influence the connection between the *c*-loop residues R45, Q46 and E48 with residues F24 and K21 at the bottom of the *Mt*εNTD, respectively. Subsequently, this interaction could translate proton-conduction via the *Mt*εNTD epitopes consisting of amino acids V51-V53, L61-A64, I8-A10, and D12-N14 to the region A81-I90 which is near to the *Mt*εCTD and includes E85. Here, amino acid F86 of the *Mt*εNTD and E87-I90 of the -CTD form a hinge region. The latter would enable up- and down movements of the C-terminal domain (Fig. 1a), resulting in a transition of a compact (ε_c_) to an extended (ε_e_) state of this subunit. Considering the hydrogen-bond interaction between E85 of the *Mt*εNTD and R113 of the -CTD, substitution of R113 to an alanine is expected to interrupt this link and altering the interaction of both domains, including the proposed sequential coupling steps described above. Interestingly, the most extensive chemical shift perturbations of the *Mt*εR113A mutant identified were A12-W16, D48-V53, K59-L70, S83–88 and S99-D121 (Fig. 7b), which encompass the residues that were reported to be important for inter-domain interactions, namely D47-A49, E87-D91, and A108-V117, as well as those from the BDQ-binding epitope, A10-W16^10^. The latter sheds light on the observation that the *M. smegmatis* ε^4A^ mutant showed a 3-fold higher MIC_50_ for BDQ compared to WT bacteria (Fig. S3a). Furthermore, the interruption of E85 and R113, leading to extensive chemical shift perturbations of the residues D48-V53 and S99-D121, would change the recently described inter-domain interactions between the C-terminal chains of A108 and A112 with D47-A49^10^, and finally the regulation of the C-terminal up- and down movements.

The R-to-A substitutions are not only affecting the interactions at the interface between the NTD and CTD of mycobacterial subunit ε but also the interaction of its CTD with the C-termini of the α_3_:β_3_-domain. Recent crosslinking studies demonstrated the close neighborhood of residue D121 of *Mt*ε with the DELSEED-region of α^10^^.^ Together with a structural model, these data led to a proposed arrangement of R113 in helix α2 of *Mt*ε to come in vicinity to another α-subunit via V117, thereby contributing to a connection with the DELSEED-part (Fig. 1a). Furthermore, R115 of helix α2 of subunit ε comes in vicinity to the DELSEED-part. At the same time, two β-subunits are proposed to interact with helix α2 of *Mt*ε. These interactions of *Mt*ε with the C-termini of α-β would provide sufficient contact to ATP synthesis and hydrolysis inside the catalytic α_3_:β_3_-headpiece. These connections would be disrupted in the case of IMVs of the *M. smegmatis* ε^4A^ mutant, causing a drastic drop of ATP synthesis (Fig. 5a). An inhibitory effect caused by the mycobacterial subunit ε would support recent data of the IMVs of the *M. smegmatis* ε_1–120_ mutant, with a deletion of the very C-terminal amino acid D121^10^. In comparison, mutants defective of the very 10^35^, 45^36^ or 51^37^ C-terminal residues of the *E. coli* subunit ε displayed no significant difference in cell growths or ATP synthesis, highlighting the structural, mechanistic and regulatory specificities of the mycobacterial protein.

As shown by solution NMR, introduction of R-to-A substitutions into the recombinant *Mt*ε (*Mt*εR113A, *Mt*εR111A,R113A, *Mt*εR111A, R113A,R115A, and *Mt*εR105A,R111A, R113A,R115A) disrupts the interactions at the interface between the NTD and CTD that are critical to its structural integrity (Fig. 7a–c). While the loss of contact upon mutating R113 may possibly be alleviated by nearby arginine residues, albeit marginally, substituting all arginine residues results in the unravelling of the protein, which manifests as oligomers. Considering the genes for the enzyme complex are arranged in an operon, and the fact that subunit ε as well as similar amounts of entire F-ATP synthase were detected within the IMVs of WT and the *M. smegmatis* ε^4A^ mutant (Fig. 5b), the oligomerization of recombinant *Mt*εR111A,R113A,R115A, and *Mt*εR105A, R111A,R113A,R115A may not occur *in vivo* (Figs 6, S5a,b). However, the R-to-A substitutions are enough to interrupt dramatically the function of the molecular engine in synthesis direction and to a lesser degree, to a reduction (40%) in hydrolysis of ATP. These data reflect that ATP synthesis and hydrolysis are not simply reverse mechanisms.

The presented data showing that genetic disruption of ε’s coupling function causes substantial loss of ATP synthesis activity, due to uncoupled H^+^-transduction, provided valuable information pertaining to the potential of this subunit as drug target for TB drug discovery. This is supported by unravelling EGCG as a novel inhibitor against F-ATP synthases of both fast- and slow growing mycobacteria. EGCG has reported activity against TB^38,39^ and was described to impact the integrity of the mycobacterial cell wall^40^. Since the ε^4A^ mutant displayed changes in cell size, morphology and meropenem sensitivity, reflecting alterations in the cell envelope and bacterial surface properties of the mutant strain, which is related to ATP reduction, the data presented indicate, that EGCG binds to ε of the mycobacterial F-ATP synthase and disrupts the crosstalk between ε’s NTD and CTD with consequences in ATP- and cell wall formation. Its novel mechanism of action reflects the spring-like coupling mechanism of the central mycobacterial ε subunit, described recently^10^. These novel insights also fittingly complement the previous studies that established the binding of BDQ to mycobacterial subunit ε^10,41,42^. By inhibiting ATP synthesis of the BDQ-resistant *M. smegmatis* F-ATP synthase mutant I66M, the current work and the unravelled EGCG pave the way for additional options for inhibiting ATP synthesis in *M. tuberculosis*. Finally, this work supports metabolism as a mediator of tolerance^42^. Metabolic reprogramming of mycobacteria as shown by the increased killing of *M. tuberculosis* by BDQ when grown on non-fermentable energy sources, affects bactericidal activity of the drug and thereby antibiotic tolerance^43^.

## Methods

The detailed descriptions of all methods can be found in Supporting Information.

### Bacterial strains and growth condition

In brief, *M. smegmatis* mc^2^ 155 (ATCC 700084) was used in this study as the parental strain. For standard cultivation, all mycobacterial strains were grown at 37 °C in Middlebrook 7H9 broth or on Middlebrook 7H10 agar plates unless otherwise stated, and antibiotics were added to the culture media as needed. For growth curve in 7H9, mid-log-phase pre-cultures (OD_600_ = 0.4–0.6) were diluted to OD_600_ of 0.05 and OD was measured at various time points until the cultures reached stationary phase. Smear for each strain was made from mid-log-phase pre-cultures and acid-fast stained using a TB stain kit (BD, 212520) according to manufacturer’s instructions. The minimal media used in the study were either prepared as previously described^44^ or as Hartmans-de Bont (HdB) media^45,46^. 0.2% glycerol, glucose or sodium acetate was added as a sole carbon source, and antibiotics were added to the culture media as needed. For growth curve in minimal media, pre-cultures were diluted to OD_600_ of 0.005 and OD was measured at various time points until the cultures reached stationary phase.

### Construction of the *M. smegmatis* atpC(R105A,R111A,R113A,R115A) mutant and the complemented strain

The detailed descriptions of methods can be found in Supporting Information. To generate a quadruple mutant with substitutions of arginine codons to alanine codons in the gene *atpC*: R105A, R111A, R113A and R115A (ε^4A^ mutant) in *M. smegmatis* F-ATP synthase, site-directed genomic mutagenesis by recombineering was carried out as described previously^42^. The final 1,219 kb double-stranded DNA (dsDNA) oligonucleotide that was used in recombineering was created in two steps using PCR, and then transformed into electro-competent *M. smegmatis* mc^2^ 155, which harbours the plasmid pJV53 that express mycobacteriophage Che9c recombineering genes gp60 and gp61, both being necessary for dsDNA homologous recombination. The obtained transformants were screened by MAMA (mismatch amplification mutation assay) colony PCR and a final verification of all four mutations in a single mutant (ε^4A^ mutant) was done by DNA sequencing. For complementation, a plasmid pMV262 containing the WT allele of *atpC* was electroporated into the ε^4A^ mutant of *M. smegmatis*. The complemented strain: ε^4A^ mutant/pMV262-*atpC* was confirmed by PCR and this plasmid was maintained in media containing kanamycin.

## Supplementary information

Supplementary Information file

## References

[1] Wayne, L. G. & Kubica, G. P. In *Bergey’s manual of systematic bacteriology* Vol. 2 (ed. Peter H. A. Sneath) 1435–1457 (Williams & Wilkins, 1986).

[2] Cook, G. M., Hards, K., Vilchèze, C., Hartman, T. & Berney, M. Energetics of respiration and oxidative phosphorylation in mycobacteria. *Microbiol. Spectr*. **2**, 10.1128/microbiolspec.MGM2-0015-2013 (2014). 10.1128/microbiolspec.MGM2-0015-2013 PMC4205543 25346874

[3] Sassetti CM, Boyd DH, Rubin EJ (2003). Genes required for mycobacterial growth defined by high density mutagenesis. Mol. Microbiol..

[4] Andries K (2005). A Diarylquinoline Drug Active on the ATP Synthase of *Mycobacterium tuberculosis*. Science.

[5] Andries K (2014). Acquired resistance of *Mycobacterium tuberculosis* to bedaquiline. PLoS One.

[6] Hartkoorn RC, Uplekar S, Cole ST (2014). Cross-resistance between clofazimine and bedaquiline through upregulation of MmpL5 in *Mycobacterium tuberculosis*. Antimicrob. Agents Chemother..

[7] Zhang Alice Tianbu, Montgomery Martin G., Leslie Andrew G. W., Cook Gregory M., Walker John E. (2019). The structure of the catalytic domain of the ATP synthase fromMycobacterium smegmatisis a target for developing antitubercular drugs. Proceedings of the National Academy of Sciences.

[8] Lu P, Lill H, Bald D (2014). ATP synthase in mycobacteria: Special features and implications for a function as drug target. Biochim. Biophys. Acta.

[9] Preiss L (2015). Structure of the mycobacterial ATP synthase F_o_ rotor ring in complex with the anti-TB drug bedaquiline. Sci Adv.

[10] Joon S (2018). The NMR solution structure of *Mycobacterium tuberculosis* F-ATP synthase subunit ε provides new insight into energy coupling inside the rotary engine. FEBS J..

[11] Cingolani G, Duncan TM (2011). Structure of the ATP synthase catalytic complex (F_1_) from *Escherichia coli* in an autoinhibited conformation. Nat. Struct. Mol. Biol..

[12] Haagsma AC, Driessen NN, Hahn M-M, Lill H, Bald D (2010). ATP synthase in slow- and fast-growing mycobacteria is active in ATP synthesis and blocked in ATP hydrolysis direction. FEMS Microbiol. Lett..

[13] Hotra A (2016). Deletion of a unique loop in the mycobacterial F-ATP synthase γ subunit sheds light on its inhibitory role in ATP hydrolysis-driven H+ pumping. FEBS J..

[14] Ragunathan P (2017). The uniqueness of subunit α of mycobacterial F-ATP synthases: An evolutionary variant for niche adaptation. J. Biol. Chem..

[15] Ferguson SA, Cook GM, Montgomery MG, Leslie AGW, Walker JE (2016). Regulation of the thermoalkaliphilic F_1_-ATPase from *Caldalkalibacillus thermarum*. Proc. Natl. Acad. Sci. USA.

[16] Uhlin U, Cox GB, Guss JM (1997). Crystal structure of the ϵ subunit of the proton-translocating ATP synthase from *Escherichia coli*. Structure.

[17] Wilkens S, Capaldi RA (1998). Solution structure of the ε subunit of the F_1_-ATPase from *Escherichia coli* and interactions of this subunit with β subunits in the complex. J. Biol. Chem..

[18] Yagi H (2007). Structures of the thermophilic F_1_-ATPase ε subunit suggesting ATP-regulated arm motion of its C-terminal domain in F_1_. Proc. Natl. Acad. Sci. USA.

[19] Lamprecht DA (2016). Turning the respiratory flexibility of *Mycobacterium tuberculosis* against itself. Nat. Commun..

[20] Dick T, Lee BH, Murugasu-Oei B (1998). Oxygen depletion induced dormancy in *Mycobacterium smegmatis*. FEMS Microbiol. Lett..

[21] Viswanathan G, Yadav S, Raghunand TR (2017). Identification of Mycobacterial Genes Involved in Antibiotic Sensitivity: Implications for the Treatment of Tuberculosis with β-Lactam-Containing Regimens. Antimicrob. Agents Chemother..

[22] Bogdanović N (2018). Structure and function of Mycobacterium-specific components of F-ATP synthase subunits α and ε. J. Struct. Biol..

[23] Vuataz L, Brandenberger H, Egli RH (1959). Plant phenols: I. Separation of the tea leaf polyphenols by cellulose column chromatography. J. Chromatogr..

[24] Kundu, S. *Bedaquiline targets the epsilon subunit of mycobacterial F-ATP synthase*. PhD thesis, National University of Singapore, (2017).

[25] Berney M, Cook GM (2010). Unique flexibility in energy metabolism allows mycobacteria to combat starvation and hypoxia. PLoS One.

[26] Wu M-L, Dick T (2015). Metabolic flexibility and morphological plasticity in mycobacteria. Future Microbiol..

[27] Tran SL, Cook GM (2005). The F_1_F_o_-ATP synthase of *Mycobacterium smegmatis* is essential for growth. J. Bacteriol..

[28] Gengenbacher M, Kaufmann SHE (2012). *Mycobacterium tuberculosis*: success through dormancy. FEMS Microbiol. Rev..

[29] Marrero J, Rhee KY, Schnappinger D, Pethe K, Ehrt S (2010). Gluconeogenic carbon flow of tricarboxylic acid cycle intermediates is critical for *Mycobacterium tuberculosis* to establish and maintain infection. Proc. Natl. Acad. Sci. USA.

[30] Fisher MA, Plikaytis BB, Shinnick TM (2002). Microarray analysis of the *Mycobacterium tuberculosis* transcriptional response to the acidic conditions found in phagosomes. J. Bacteriol..

[31] Eoh H, Rhee KY (2013). Multifunctional essentiality of succinate metabolism in adaptation to hypoxia in *Mycobacterium tuberculosis*. Proc. Natl. Acad. Sci..

[32] Hards K (2015). Bactericidal mode of action of bedaquiline. J. Antimicrob. Chemother..

[33] Hards K (2018). Ionophoric effects of the antitubercular drug bedaquiline. Proc. Natl. Acad. Sci..

[34] Kaguni JM (2006). DnaA: Controlling the initiation of bacterial DNA replication and more. Annu. Rev. Microbiol..

[35] Kuki M, Noumi T, Maeda M, Amemura A, Futai M (1988). Functional domains of epsilon subunit of *Escherichia coli* H^+^-ATPase (F_0_F_1_). J. Biol. Chem..

[36] Shah NB, Duncan TM (2015). Aerobic growth of *Escherichia coli* is reduced, and atp synthesis is selectively inhibited when five C-terminal residues are deleted from the ϵ subunit of ATP synthase. J. Biol. Chem..

[37] Taniguchi N, Suzuki T, Berney M, Yoshida M, Cook GM (2011). The regulatory C-terminal domain of subunit ε of F_o_F_1_ ATP synthase is dispensable for growth and survival of *Escherichia coli*. J. Bacteriol..

[38] Anand PK, Kaul D, Sharma M (2006). Green tea polyphenol inhibits *Mycobacterium tuberculosis* survival within human macrophages. Int. J. Biochem. Cell Biol..

[39] Soh Avril, Pan An, Chee Cynthia, Wang Yee-Tang, Yuan Jian-Min, Koh Woon-Puay (2017). Tea Drinking and Its Association with Active Tuberculosis Incidence among Middle-Aged and Elderly Adults: The Singapore Chinese Health Study. Nutrients.

[40] Sun T (2015). Effects of epigallocatechin gallate on the cell-wall structure of *Mycobacterial* smegmatis mc2155. Nat. Prod. Res.

[41] Biuković G (2013). Variations of subunit ε of the *Mycobacterium tuberculosis* F_1_F_o_ ATP synthase and a novel model for mechanism of action of the tuberculosis drug TMC207. Antimicrob. Agents Chemother..

[42] Kundu S, Biukovic G, Grüber G, Dick T (2016). Bedaquiline targets the ε subunit of mycobacterial F-ATP synthase. Antimicrob. Agents Chemother..

[43] Koul A (2014). Delayed bactericidal response of *Mycobacterium tuberculosis* to bedaquiline involves remodelling of bacterial metabolism. Nat. Commun..

[44] van Kessel JC, Marinelli LJ, Hatfull GF (2008). Recombineering mycobacteria and their phages. Nat. Rev. Microbiol..

[45] Hartmans S, De Bont JA (1992). Aerobic vinyl chloride metabolism in *Mycobacterium aurum* L1. Appl. Environ. Microbiol..

[46] Smeulders MJ, Keer J, Speight RA, Williams HD (1999). Adaptation of *Mycobacterium smegmatis* to stationary phase. J. Bacteriol..

[47] Sobti M (2016). Cryo-EM structures of the autoinhibited *E. coli* ATP synthase in three rotational states. eLife.

