# Supplementary material for: Disrupting coupling within mycobacterial F-ATP synthases subunit ε causes dysregulated energy production and cell wall biosynthesis
Source: Sci Rep. 2019 Nov 14;9:16759. doi: 10.1038/s41598-019-53107-3 (PMC6856130; doi:10.1038/s41598-019-53107-3)
Supplement: Supplementary file 1 — Supplementary Information file [file 41598_2019_53107_MOESM1_ESM.pdf]

Disrupting coupling within mycobacterial F-ATP synthases subunit ε causes dysregulated energy production and cell wall biosynthesis

Wuan-Geok Saw[1,#], Mu-Lu Wu[2,#], Priya Ragunathan[1,#], Goran Biuković[2], Aik-Meng Lau[1], Joon Shin[1], Amaravadhi Harikishore[3], Chen-Yi Cheung[4], Kiel Hards[4], Jickky Palmae Sarathy[2], Roderick Bates[3], Gregory M. Cook[4], Thomas Dick[2,5,]* and Gerhard Grüber[1,]*

[1]Nanyang Technological University, School of Biological Sciences, 60 Nanyang Drive, Singapore 637551, Republic of Singapore

[2]Department of Microbiology and Immunology, Yong Loo Lin School of Medicine, National University of Singapore, 14 Medical Drive, Singapore 117599, Republic of Singapore

[3]Division of Chemistry and Biological Chemistry, School of Physical and Mathematical Sciences, Nanyang Technological University, 21 Nanyang Link, Singapore 637371, Republic of Singapore

[4]Department of Microbiology and Immunology, School of Biomedical Sciences, University of Otago, Dunedin 9054, New Zealand

[5]Center for Discovery and Innovation, Hackensack Meridian Health, 340 Kingsland Street Building 102, Nutley, NJ 07110, USA

[#]Authors have equal contribution

*To whom correspondence should be addressed: Prof. Dr. Thomas Dick, Tel.: (+1) 201 880 3530; Email: thomas.dick@hmh-cdi.org;; Prof. Dr. Gerhard Grüber, Tel.: (65) 6316 2989, E-mail: ggrueber@ntu.edu.sg

Supplementary Information

Biochemicals

Kapa HiFi DNA polymerase was purchased from KAPA Biosystems (UK) and Ni^{2+}-NTA chromatography resin was obtained from Qiagen (Hilden, Germany). Phusion DNA polymerase was purchased from Thermofisher Scientific. Enzymatic digestion was performed using restriction enzymes from New England BioLabs. Chemicals from Bio-Rad (California, USA) were used for gel electrophoresis. All other chemicals of analytical grade were obtained from Biomol (Hamburg, Germany), Merck (Darmstadt, Germany), Sigma, or Serva (Heidelberg, Germany). Primers and dsDNA oligonucleotides were synthesized by Integrated DNA Technologies (USA). The ATP levels were measured using cell titer glow from Promega (Madison, Wisconcin, USA).

Bacterial strains and growth conditions

M. smegmatis mc^2 155 (ATCC 700084) was used in this study as the parental strain. For standard cultivation, all mycobacterial strains were grown at 37 °C in Middlebrook 7H9 broth (supplemented with 0.5% bovine albumin, 0.2% glucose, 0.085% NaCl, 0.5% glycerol, 0.0003% catalase, and 0.05% Tween80) or on Middlebrook 7H10 agar plates (supplemented with 0.5% bovine albumin, 0.2% glucose, 0.085% NaCl, 0.5% glycerol, 0.0003% catalase, and 0.006% oleic acid) unless otherwise stated. When required, antibiotics were added to the culture media (broth or agar) for selection at the following final concentrations: kanamycin 25 µg/ml; hygromycin 50 µg/ml. For growth curve in 7H9, mid-log-phase pre-cultures (OD_{600} = 0.4-0.6) were diluted to OD_{600} of 0.05 and OD was measured at various time points until the cultures reached stationary phase.

Minimal media were either prepared as previously described[1] with slight modification (6 g/l Na_2HPO_4, 3 g/l KH_2PO_4, 0.5 g/l NaCl, 1 g/l NH_4Cl, 2 mM $MgSO_4$, 0.1 mM $CaCl_2$, 0.05% Tyloxapol, pH 7.2-7.4) or as Hartmans de Bont (HdB) media[2,3] supplemented with 0.05% Tween 80. 0.2% glycerol or sodium acetate was added as a sole carbon source. For initial starter cultures, strains were inoculated from 7H10 agar plate into HdB minimal media with the corresponding carbon source. For complemented strain, 25 μg/ml of kanamycin was added. The starter cultures were grown at 37 °C for 48 hrs. After that, the starter cultures were diluted to OD_{600} of 0.005 in minimal media with the corresponding carbon source. All strains were grown in baffled flask at 37 °C with agitation (120 rpm).

Construction of the *M. smegmatis* atpC(R105A,R111A,R113A,R115A) mutant and the complemented strain

To generate a quadruple mutant with substitutions of arginine codons to alanine codons in the gene *atpC*: R105A, R111A, R113A and R115A (ε^{4A} mutant) in *M. smegmatis* mc^2 155 F-ATP synthase, site-directed genomic mutagenesis by recombineering was carried out as described previously[4]. The final 1,219 kb double-stranded DNA (dsDNA) oligonucleotide that was used in recombineering was created in two steps: first, the upstream 586 bp DNA homology and the downstream 533 bp DNA homology of the *atpC* gene were amplified in two separate PCR reactions (PCRA and PCRB) using genomic DNA of *M. smegmatis* mc^2 155 as a template and the following primers: PCRA-Ext-Forward 5'-CGC GAT CCT CGG TAT CGA CGA GC-3'/ PCRA-Ext-Reverse 5'-CGG ATG CTG CGT CTT CCT TGG CA-3' and PCRB-Ext-Forward 5'-GCG CCC TCG GCC AGA TCG ACT AG-3'/ PCRB-Ext-Reverse 5'-CGA AGT ACC TCA ACC GGC TGT CGG AC-3'.

Second, both PCR products were used as a template along with the 100 bp dsDNA synthesized oligonucleotide containing quadruple mutation (R105A, R111A, R113A, and R115A): 5'-GCT GCC AAG GAA GAC GCA GCA TCC GAC GAC GAG GCG ACC GCG GCA TGG GGC GCC GCG GCG CTG GCG GCC CTC GGC CAG ATC GAC TAG TCA CAT CGA TGA G-3' to generate a final 1,219 kb recombineering dsDNA fragment. This recombineering dsDNA fragment was used in transformation of the electro-competent cells of *M. smegmatis* mc^2 155, harbouring the plasmid pJV53 and expressing mycobacteriophage Che9c recombineering genes gp60 and gp61, both being necessary for dsDNA homologous recombination. The obtained transformants were screened by MAMA (mismatch amplification mutation assay) colony PCR using the following primers: 4R-Forward 5'-GGC TGA TCT GAA CGT CGA GAT CGT CGC-3'/ 4R-Reverse 5'-ATA GTC CCG CAG GAT CGC CGC C-3'. A final verification of all four mutations in a single mutant (ε^{4A} mutant) was done by DNA sequencing.

For complementation, a plasmid pMV262 containing the WT allele of *atpC* under the hsp60 promoter[1] was electroporated into the ε^{4A} mutant of *M. smegmatis* mc^2 155. The complemented strain: ε^{4A} mutant/pMV262-*atpC* was confirmed by PCR using a primer set, which binds to the hsp60 promoter and *atpC* gene, and giving an amplicon for the complemented strain only. An ε^{4A} mutant/pMV262-atpC was always maintained in media containing kanamycin to keep the episomal plasmid unless otherwise stated. The ε^{4A} mutant containing empty plasmid pMV262 was used throughout the experiments as a negative control and it behaves identical to ε^{4A} mutant (data not shown).

DNA isolation and whole genome sequencing

10 ml of mid-log-phase cultures of WT *M. smegmatis* mc^2 155 and the ε^{4A} mutant strain grown in Middlebrook 7H9 broth were collected and resuspended in lysis buffer containing 10 mM Tris/HCl, pH 8, 100 mM NaCl, 3% SDS, 1 mM $CaCl_2$ and 0.2 mg proteinase K. The samples were incubated at 56 °C for 30 min, and the cells were mixed with zirconia beads and lysed with vortex mixer. After removing the cell debris by centrifugation, 2 mM EDTA was added into each sample, and mixed vigorously after adding 1 volume of phenol:chloroform:isoamyl alcohol (25:24:1). After centrifugation, the aqueous layer was transferred into a new tube, and mixed with 0.1 volume of 3 M sodium acetate (pH 5.2) and 0.8 volume of isopropanol. After centrifugation, the DNA pellet was washed twice with 700 µl of ice-cold 70% ethanol. After centrifugation and air drying, the DNA pellet was resuspended in HyClone™ water, and the genomic DNA was quality checked with gel electrophoresis.

The whole genome sequencing (WGS) of the isolated genomic DNA of both strains was performed at NovogeneAIT Genomic Singapore. The DNA library was constructed at the Beijing Novogene Bioinformatics Technology Co. Ltd, and the WGS was performed on the Illumina platform with MPS (massively parallel sequencing) Illumina technology. The adapter reads and low-quality reads from the paired-end and mate-pair library were filtered using the pipeline compiled by NovogeneAIT. All good quality paired reads were then assembled into a few scaffolds using the program SOAPdenovo[5,6].

The fasta file of *M. smegmatis* mc^2 155 genomic sequence (GenBank: NC_008596) was retrieved from NCBI and used as reference genome. The good quality paired reads were first mapped to the reference genome using Bowtie2 (v2.3.5.1)[7]. The resulting SAM file was then converted into a BAM file, sorted and indexed using SAMtools software package (v1.7)[8]. The

SAMtools mpileup[9] was used to generate mpileup file for the variant calling using VarScan (v2.3.9)[10] with mpileup2snp and mpileup2indel, with the p-value threshold of 0.05. Then the generated vcf files of WT and the ε^{4A} mutant were viewed and compared with the reference genome using Integrative Genomics Viewer (IGV v2.6.2). The gene functions of the mutation sites were identified on NCBI database. Only nonsynonymous mutations, insertions and deletions that satisfying the threshold criteria (Quality > 30, frequency > 75%, coverage > 20) from the mutant strain and not found in the WT strain were included in the final analysis.

Molar growth yields and biochemical assays

The molar growth yields on glycerol ($Y_{glycerol}$) (in grams [dry weight] of cells per mol of glycerol consumed) or acetate ($Y_{acetate}$) (in grams [dry weight] of cells per mol of acetate consumed) were determined at stationary phase (48 or 72 h) using the procedures as described previously[11]. To determine the dry weight of each strain, 50 ml of culture was harvested in triplicate by filtration (0.22 μm; Millipore), and filters were dried at 65°C until the same weight (dry weight) was achieved on consecutive days. To determine the consumption of glycerol or acetate at each time point, supernatant samples after filtration were collected after the initial inoculation to determine the starting glycerol/acetate concentration of each individual flask and at specified time points during growth. Glycerol or acetate concentration was measured by detecting NADH oxidation (absorbance at 340 nm) using previously published methods[12]. Briefly, a working solution of assay mix contained 0.5 U/ml pyruvate kinase from rabbit muscle (Sigma-Aldrich), 1 U/ml lactic dehydrogenase from rabbit muscle (Roche), 50 mM Tris-HCl (pH 8), 2 mM $MgCl_2$, 0.25 mM NADH, 3 mM phosphoenolpyruvate (PEP) and 3 mM ATP. Depend on the carbon source, 0.5 U/ml glycerokinase from *E. coli* (Sigma-Aldrich) or 1 U/ml acetate

kinase from *E. coli* (Sigma-Aldrich) was added into assay mix. A standard curve was prepared from 13.7 mM glycerol stock, ranged from 0 to 136.8 μM, or 1 M acetate stock, ranged from 0 to 0.732 mM. The reaction mixtures were incubated in microcentrifuge tubes for 15 min at 37 °C to allow the enzyme assay to reach an endpoint. Biological replicates were performed 3 times for each time point.

Colony and cell morphology of mycobacterial strains

Pre-cultures were grown to mid-log-phase and diluted to OD_{600} of 0.1 and further diluted to 10^{-5}. 50 μl of the diluted cultures were plated on 7H10 agar plates and incubated at 37 °C for 3 days. Pictures of isolated single colonies were taken using a Nikon SMZ-745T stereomicroscope connected to a Nikon DS-Fi2 digital camera and a DS-L3 camera controller.

Smear for each strain was made from mid-log-phase pre-cultures and acid-fast stained using a TB stain kit (BD, 212520) according to manufacturer's instructions. Bacteria cell morphology was observed under bright field of Nikon Eclipse Ni-U microscope. Cell length was measured using ImageJ.

OCR and ECAR Measurements

Extracellular flux analysis was performed in a fluorespirometer (OROBOROS Oxygraph-2k, Innsbruck, Austria). OCR and ECAR were measured simultaneously by both clark type electrode[13] and fluorescence intensity of the pH responsive probe pyranine[14]. Prior to the experiment, cells were grown on 7H9 Medium supplemented with ADS and 0.05% Tween 80. Whole cells of *M. smegmatis* (OD_{600} = 1.0) were washed and resuspended in a modified Hartmans de Bont, containing no phosphate buffer or carbon source, henceforth referred to as

Mycobacterial Respiration buffer A (MRA). The composition of MRA per litre is 9.2 g NaCl, 0.4 g KCl, 2 g $(NH_4)_2SO_4$, 10 ml 10x trace metal solution[15], adjusted to pH 7.2 with 100 mM HCl or NaOH as necessary. Pyranine (Sigma, 20 µM) was added to the washed cell suspension prior to the experiment. Pyranine fluorescence was calibrated to pH as previously described[14], with the exception that only one fluorescence wavelength is measured by the Oroboros fluorespirometer. In this regard, the Oroboros Blue LEDs (465 nm) were fitted with the manufacturer supplied safranin LED filter and magnesium green photodiode filter. Gain was set to 10 and intensity was set to 700. For MRA the calibration curve was linear between pH 6.0 and 8.5, with the equation: V = 0.6596*pH - 3.708 (R^2 = 0.9656, number of data points = 24), where V = voltage response of the photodiode. Before each experiment, the oxygen concentration in the medium was equilibrated for 2–3 min with air until a stable signal was obtained at an oxygen concentration of approximately 220 µM. The stoppers were subsequently inserted and the pH was adjusted to 7.0-7.2 with NaOH. Chemicals were added through the injection port of the stoppers using Hamilton syringes. All measurements were made at 37 °C with 750 rpm and a data recording interval of 1 s^{-1}.

BDQ and meropenem susceptibility test

500 µM stock of BDQ was prepared in 100% DMSO. An 8-point 2-fold serial dilution was carried from 150 nM to 1.17 nM. 500 µl of each concentration was aliquoted into a 14 ml round bottom tube and mixed with 500 µl of OD_{600} = 0.1 cultures diluted from mid-log-phase pre-cultures. The final concentration range for BDQ is from 75 nM to 0.59 nM and the final OD_{600} for mycobacterial cultures is 0.05. Tubes were incubated at 37 °C for 24 h with orbital shaking at 160 rpm, and then OD_{600} was measured. MIC_{50} was defined as the concentration that inhibits

50% of growth compared to the drug-free control. For the ε^{4A} complementary strain, pre-cultures were centrifuged and resuspended in fresh 7H9 broth to remove kanamycin in the pre-cultures and no kanamycin was added during the susceptibility test, as the presence of kanamycin would affect BDQ MIC_{50}.

The meropenem susceptibility assay was carried out using the broth microdilution method as described previously[16]. Briefly, clear 96-well flat-bottom Costar cell culture plates (Corning) were filled with 100 µl of complete 7H9 medium in each well. Meropenem was added to the first well in each row to create two times the desired highest final concentration. Subsequently, a 10-point 2-fold serial dilution was carried out starting from the first well in each row, with the final concentration range for meropenem from 100 µM to 0.1 µM. Cultures of *M. smegmatis* WT, ε^{4A} mutant and ε^{4A} complement mutant were grown to mid-log phase and subsequently diluted to an OD_{600} of 0.1. 100 µl of the diluted culture was added to each well to create a final OD_{600} of 0.05 in all wells. The plates were incubated at 37°C without agitation for 1 day. At the end of the incubation period, the cultures in all wells were resuspended and the OD_{600} was read using a TECAN Infinite Pro 200 plate reader. The MIC_{50} reported represents the concentration that inhibits 50% of growth compared to the untreated culture.

Determination of intracellular ATP level

8 ml of mid-log-phase cultures of each strain grown in minimal media containing 0.2% sodium acetate were collected and washed once in Phosphate-buffered-saline (PBS) to remove extracellular ATP and resuspended in 800 µl PBS. From which 50 µl was taken out to measure the ATP level using the BacTiter-Glo Microbial Cell Viability Assay (Promega) as previously described[17]. The remaining was transferred to 2 ml Lysing Matrix B tubes and disrupted by

FastPrep-24TM 5G Instrument (MP Biomedicals) to extract total protein. After centrifuge, protein concentrations in the supernatant were determined using PierceTM BCA Protein Assay Kit (Thermo Fisher Scientific) according to manufacturer's instructions. ATP content in each sample was calculated based on ATP standard solutions ranging from 0.1 nM to 1 µM done in each experiment.

2 ml of stationary-phase cultures of each strain grown in minimal media containing 0.2% glucose were collected and washed once in Phosphate-buffered-saline (PBS) to remove extracellular ATP and resuspended in 200 µl PBS. From which 75 µl were taken out to measure the ATP level using the CellTiter-Glo® Luminescent Cell Viability Assay (Promega). The concentration of the cells was estimated by measuring the remaining sample at OD_{600}. The luminescent signal of each sample was then normalized with their corresponding OD_{600}. To compare the difference between WT *M. smegmatis* and the mutant, the data were further normalized according to the value of WT.

Preparation of inverted membrane vesicles from *M. smegmatis*

In order to purify IMVs of *M. smegmatis* for ATP synthesis, and -hydrolysis assays, cells were cultivated overnight at 37 °C in 7H9 supplemented with 10% ADC, 0.5% glycerol and 0.05% Tween80 until it reached OD_{600} 0.6-0.7. The culture was expanded in 200 ml supplemented 7H9 and grown in 1 litre shake flasks (180 rpm) until OD_{600} 0.6-0.7. This culture was used to inoculate a 500 ml culture that was grown overnight in 2 litre shake flasks (180 rpm) until an OD_{600} of 0.6-0.7. About 5 g (wet weight) of WT *M. smegmatis* and mutant ε^{4A} were resuspended in 20 ml membrane preparation buffer (50 mM MOPS, 2 mM $MgCl_2$, pH 7.5) containing EDTA-free protease inhibitor cocktail (1 tablet in 20 ml buffer, Roche-USA) and 1.2

mg/ml lysozyme. The suspension was stirred at room temperature for 45 min and additionally supplemented with 15 mM $MgCl_2$ and 50 μl DNaseI (Thermo Fischer, USA), and continued stirring for another 15 min at room temperature. All subsequent steps were performed on ice. Cells were broken by three passages through an ice precooled Model M-110L Microfluidizer processor (M-110L) at 18,000 psi. The suspension containing lysed cells was centrifuged at 4,200 x g at 4 °C for 20 min. The supernatant containing membrane fraction was further subjected to ultracentrifugation 45,000 x g at 4 °C for 1 h. The supernatant was discarded, and the precipitated membrane fraction was resuspended in membrane preparation buffer containing 15% glycerol, aliquoted, snap frozen and stored at -80 °C. The concentrations of the proteins in the vesicles were determined by the BCA method. Inverted membrane vesicles were stored at -80 °C.

ATP hydrolysis and -synthesis measurements

A continuous ATP hydrolysis assay was applied to measure the ATPase activity of WT IMVs of *M. smegmatis* mc^2 155, the ε^{4A} mutant and ε^{4A} mutant complement strain. In case of IMVs the consumption of NADH by the type II NADH dehydrogenase (NDH-2) was inhibited by thioridazine. In addition, ATPase activity of IMVs was also measured in the absence of ATP and presence of thiorodazine to identify any background NADH oxidation and MgATP hydrolysis. ATP synthesis was measured as described previously[18].

Assay for proton translocation

Proton translocation of IMVs of *M. smegmatis* with WT F-ATP synthase or the ε^{4A} mutant was measured by a decrease of 9-amino-6-chloro-2-methoxyacridine (ACMA) fluorescence using a Cary Eclipse Fluorescence spectrophotometer (Varian Inc., Palo Alto)[18].

Western blot analysis

M. smegmatis IMVs of WT, the ε^{4A} mutant, the ε^{4A} complemented mutant or the recombinant *Mt*ε were fractionated by a 12% SDS-gel and transferred to a nitrocellulose membrane using a semi-dry transfer apparatus according to the manufacturer's protocols (Carl Roth, Karlsruhe). After blocking with 3% gelatin in a TBSN-Tween buffer (20 mM Tris-HCl, pH 7.5, 500 mM NaCl, 0.02% NaN_3 and 0.05% Tween20) for 1 h at room temperature, the membrane was washed with 1% gelatin in TBSN-Tween for 3 x 10 min. Afterwards, the membranes were incubated with rabbit antiserum directed against subunit β or -ε of *E. coli* F_1F_O ATP synthase (1:2,000) diluted with 1% gelatin TBSN-Tween for 1 hour at room temperature, followed by three washing steps (15 min) with TBSN-Tween buffer. The antibodies were kindly provided by Professor M. Futai and Professor M. Nakanishi (Department of Biochemistry, Faculty of Pharmaceutical Sciences, Iwate Medical University, Japan). The membranes were incubated with a 1:30,000 dilution of alkaline phosphatase-conjugated anti-rabbit antibodies (SIGMA) for one hour at room temperature, before washing the membranes three times with TBSN-Tween and development with substrate solution (Nitro blue tetrazolium (NBT) and 5-bromo-4-chloro-indolyl phosphate (BCIP) in 50 mM Tris-HCl, pH 7.5, 100 mM NaCl, 50 mM $MgCl_2$. The reaction was stopped by washing the membranes in water.

Cloning, production and purification of *Mt*ε mutants

To create the *Mt*ε mutants *Mt*εR113A, *Mt*εR111A,R113A, *Mt*εR111A,R113A, R115A, and *Mt*εR105A,R111A,R113A,R115A, the site-directed mutagenesis was performed using the In-Fusion strategy by Raman and Martin[54]. The polymerase chain reactions (PCR) were set up using the primers and templates shown in Table S1. The plasmid pET9d-*MtbAtpC*[19], which encodes the WT *M. tuberculosis atpC* gene encoding WT *Mt*ε, served as the starting template in the PCR for construct *Mt*εR113A. PCR products were treated with DpnI and transformed into chemically competent *E. coli* DH5α cells. To verify the incorporated mutations, the plasmids were extracted and sequenced. Once confirmed, these plasmids were used as template in the subsequent PCR for the next construct. This process was repeated until constructs for all four *Mt*ε mutants were obtained. The plasmids were then transformed into *E. coli* C41(DE3) cells for protein production.

In order to produce the recombinant His-tagged *Mt*ε as well as the mutants *Mt*εR113A, *Mt*εR111A,R113A, *Mt*εR111A,R113A,R115A, and *Mt*εR105A,R111A,R113A,R115A, cells were grown in kanamycin-supplemented (30 µg/ml) LB medium at 37 °C with shaking of 180 rpm, until an optical density OD_{600} of 0.6 - 0.7 was achieved. Production of both proteins was obtained by adding of isopropyl (thio)-β-D-galactopyranoside (IPTG) to a final concentration of 1 mM. Following overnight incubation at 15 °C, the cells were harvested at 8,500 x g for 13 min, 6 °C. Subsequently, they were lysed on ice by sonication for 3 x 1 min in buffer A (50 mM Tris/HCl, pH 8.5, 200 mM NaCl, 10% glycerol, 2 mM phenylmethane sulfonyl fluoride (PMSF) and 2 mM PefablocSC (4-(2-Aminoethyl) benzenesulfonyl fluoride hydrochloride) (BIOMOL)). Precipitated material was separated by centrifugation at 10,000 x g for 35 min, and the supernatant was filtered (0.45 µm; Millipore). The filtrate was incubated with 1.5 ml Ni^{2+}-NTA

resin for 1.5 h at 4 °C to isolate *Mt*ε and the respective mutants, according to Grüber et al.[55]. The His-tagged proteins were eluted with an imidazole-gradient (0 - 400 mM) in buffer A. Fractions containing *Mt*ε or its mutants were concentrated using Centricon YM-3 (3 kDa molecular mass cut-off) spin concentrators (Millipore) and subsequently applied onto a SEC column (Superdex 75 HR 10/30 column (GE Healthcare)). The eluting buffer consisted of 50 mM Tris/HCl (pH 8.5), 200 mM NaCl and 10% glycerol.

To produce ^{15}N-labelled *Mt*ε and its mutants *Mt*εR113A, and *Mt*εR105A,R111A, R113A,R115A for NMR spectroscopy, the seed culture of freshly transformed *E. coli* C41(DE3) cells was grown overnight. The culture was centrifuged at 3,000 x g for 10 min at 25 °C to pellet out the cells. The cells were washed and subsequently suspended in 1 litre M9 minimal media supplemented with $MgSO_4$, $CaCl_2$, thiamine, $FeCl_3$, trace elements and kanamycin.

NMR spectroscopy data acquisition

Samples for NMR experiments contained 0.2 - 0.3 mM protein in 50 mM Tris, pH 8.5, 200 mM NaCl, 10% glycerol, 0.01% NaN_3 and 10% D_2O. All NMR measurements were performed on a Bruker Avance 700 MHz spectrometer, equipped with a 5 mm z-axis-gradient cryogenic probe at 293K. The resonance assignments of ^{15}N and ^{1}HN were achieved based on our previously reported NMR chemical shift data of *Mt*ε (BMRB, accession code: 536131)[20].

Virtual screening

The NMR structure of *Mt*ε (PDB ID: 5YIO) was employed for docking studies. The protein was prepared by correcting bond orders, any missing atoms and energy minimized until the heavy atoms are converge to 0.3 Å rmsd using OPLS_2005[21] force field in Prep wizard module

in maestro modeling suite[22]. To prepare ligands for docking studies, we have employed property filtered dataset (polar surface area >90) from Zinc library 15[23] for clamp site screening. 2D coordinates from Interbioscreen were processed using LigPrep[22,24] application and energy minimized using default settings with MacroModel application in maestro modeling suite[22].

Molecular docking simulations for polar surface library were run through HTVS mode (High-Throughput Virtual Screening), the SP mode (Standard Precision), and XP mode using default settings. Docking grids were generated with the default settings in Glide using the R109 residue as the center of the grid box. Docking calculations were performed with Glide v10.5.014[22,25].

Molecular Dynamics (MD) was performed using Desmond program[22] to assess the stability ligand interactions at the C-terminal domain of *Mt*ε. The system was built for MD run by using predefine SPC solvation, orthorhombic 10x10x10 box and 9 Na^+-ions were added using 0.15 M NaCl concentration. The equilibrated set up was then used to run MD run for 200 nsec.

References

1 van Kessel, J. C., Marinelli, L. J. & Hatfull, G. F. Recombineering mycobacteria and their phages. *Nat. Rev. Microbiol.* **6**, 851, doi:10.1038/nrmicro2014 (2008).

2 Hartmans, S. & De Bont, J. A. Aerobic vinyl chloride metabolism in *Mycobacterium aurum* L1. *Appl. Environ. Microbiol.* **58**, 1220 (1992).

3 Smeulders, M. J., Keer, J., Speight, R. A. & Williams, H. D. Adaptation of *Mycobacterium smegmatis* to stationary phase. *J. Bacteriol.* **181**, 270 (1999).

4 Kundu, S., Biukovic, G., Grüber, G. & Dick, T. Bedaquiline targets the ε subunit of mycobacterial F-ATP synthase. *Antimicrob. Agents Chemother.* **60**, 6977-6979, doi:10.1128/aac.01291-16 (2016).

5 Li, R., Li, Y., Kristiansen, K. & Wang, J. SOAP: short oligonucleotide alignment program. *Bioinformatics* **24**, 713-714, doi:10.1093/bioinformatics/btn025 (2008).

6 Li, R. *et al. De novo* assembly of human genomes with massively parallel short read sequencing. *Genome Res.* **20**, 265-272, doi:10.1101/gr.097261.109 (2010).

7 Langmead, B. & Salzberg, S. L. Fast gapped-read alignment with Bowtie 2. *Nat. Methods* **9**, 357, doi:10.1038/nmeth.1923 (2012).

8 Li, H. *et al.* The Sequence Alignment/Map format and SAMtools. *Bioinformatics* **25**, 2078-2079, doi:10.1093/bioinformatics/btp352 (2009).

9 Li, H. A statistical framework for SNP calling, mutation discovery, association mapping and population genetical parameter estimation from sequencing data. *Bioinformatics* **27**, 2987-2993, doi:10.1093/bioinformatics/btr509 (2011).

10 Koboldt, D. C., Larson, D. E. & Wilson, R. K. Using VarScan 2 for Germline Variant Calling and Somatic Mutation Detection. *Curr Protoc Bioinformatics* **44**, 15.14.11-15.14.17, doi:10.1002/0471250953.bi1504s44 (2013).

11 McKenzie, J. L. *et al.* A VapBC toxin-antitoxin module is a posttranscriptional regulator of metabolic flux in mycobacteria. *J. Bacteriol.* **194**, 2189, doi:10.1073/pnas.1000715107 (2012).

12 Pinter, J. K., Hayashi, J. A. & Watson, J. A. Enzymic assay of glycerol, dihydroxyacetone, and glyceraldehyde. *Arch. Biochem. Biophys.* **121**, 404-414, doi:10.1016/0003-9861(67)90094-X (1967).

13 Hards, K. *et al.* Bactericidal mode of action of bedaquiline. *J. Antimicrob. Chemother.* **70**, 2028-2037, doi:10.1093/jac/dkv054 (2015).

14 Hards, K. *et al.* Ionophoric effects of the antitubercular drug bedaquiline. *Proc. Natl. Acad. Sci.* **115**, 7326, doi:10.1073/pnas.1803723115 (2018).

15 Berney, M., Weimar, M. R., Heikal, A. & Cook, G. M. Regulation of proline metabolism in mycobacteria and its role in carbon metabolism under hypoxia. *Mol. Microbiol.* **84**, 664-681, doi:10.1111/j.1365-2958.2012.08053.x (2012).

16 Dick, T., Lee, B. H. & Murugasu-Oei, B. Oxygen depletion induced dormancy in *Mycobacterium smegmatis*. *FEMS Microbiol. Lett.* **163**, 159-164, doi:10.1111/j.1574-6968.1998.tb13040.x (1998).

17 Wu, M.-L., Gengenbacher, M. & Dick, T. Mild nutrient starvation triggers the development of a small-cell survival morphotype in mycobacteria. *Front. Microbiol.* **7**, 947, doi:10.3389/fmicb.2016.00947 (2016).

18 Hotra, A. *et al.* Deletion of a unique loop in the mycobacterial F-ATP synthase γ subunit sheds light on its inhibitory role in ATP hydrolysis-driven H+ pumping. *FEBS J.* **283**, 1947-1961, doi:10.1111/febs.13715 (2016).

19 Biuković, G. *et al.* Variations of subunit ε of the *Mycobacterium tuberculosis* F_1F_o ATP synthase and a novel model for mechanism of action of the tuberculosis drug TMC207. *Antimicrob. Agents Chemother.* **57**, 168-176, doi:10.1128/aac.01039-12 (2013).

20 Joon, S. *et al.* The NMR solution structure of *Mycobacterium tuberculosis* F-ATP synthase subunit ε provides new insight into energy coupling inside the rotary engine. *FEBS J.* **285**, 1111-1128, doi:10.1111/febs.14392 (2018).

21 Harder, E. *et al.* OPLS3: A force field providing broad coverage of drug-like small molecules and proteins. *J. Chem. Theory Comput.* **12**, 281-296, doi:10.1021/acs.jctc.5b00864 (2016).

22 Schrödinger Release 2012-1: Protein Preparation Wizard; Epik, Macromodel, Maestro, LigPrep, Qikprop, Glide, Desmond (Schrödinger, LLC, New York, NY, 2012).

23 Sterling, T. & Irwin, J. J. ZINC 15 – Ligand discovery for everyone. *J. Chem. Inf. Model.* **55**, 2324-2337, doi:10.1021/acs.jcim.5b00559 (2015).

24 Madhavi Sastry, G., Adzhigirey, M., Day, T., Annabhimoju, R. & Sherman, W. Protein and ligand preparation: parameters, protocols, and influence on virtual screening enrichments. *J. Comput.-Aided Mol. Des.*, 1-14, doi:10.1007/s10822-013-9644-8 (2013).

25 Friesner, R. A. *et al.* Extra Precision Glide: Docking and scoring incorporating a model of hydrophobic enclosure for protein−ligand complexes. *J. Med. Chem.* **49**, 6177-6196, doi:10.1021/jm051256o (2006).

26 Toomey, R. E. & Wakil, S. J. Studies on the mechanism of fatty acid synthesis XV. Preparation and general properties of β-ketoacyl acyl carrier protein reductase from Escherichia coli. *Biochimica et Biophysica Acta (BBA) - Lipids and Lipid Metabolism* **116**, 189-197, doi:10.1016/0005-2760(66)90001-4 (1966).

27 Berney, M., Greening, C., Hards, K., Collins, D. & Cook, G. M. Three different [NiFe] hydrogenases confer metabolic flexibility in the obligate aerobe Mycobacterium smegmatis. *Environ. Microbiol.* **16**, 318-330, doi:10.1111/1462-2920.12320 (2014).

28 The UniProt, C. UniProt: a worldwide hub of protein knowledge. *Nucleic Acids Res.* **47**, D506-D515, doi:10.1093/nar/gky1049 (2018).

29 Mallette, E. & Kimber, M. S. Structure and Kinetics of the S-(+)-1-Amino-2-propanol Dehydrogenase from the RMM Microcompartment of *Mycobacterium smegmatis*. *Biochemistry* **57**, 3780-3789, doi:10.1021/acs.biochem.8b00464 (2018).

(a)

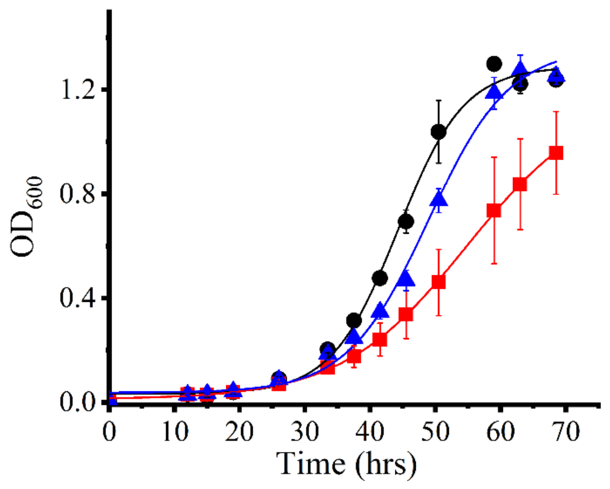

1.2
0.8
0.4
0.0
OD_{600}
0
10
20
30
40
50
60
70
Time (hrs)

(b)

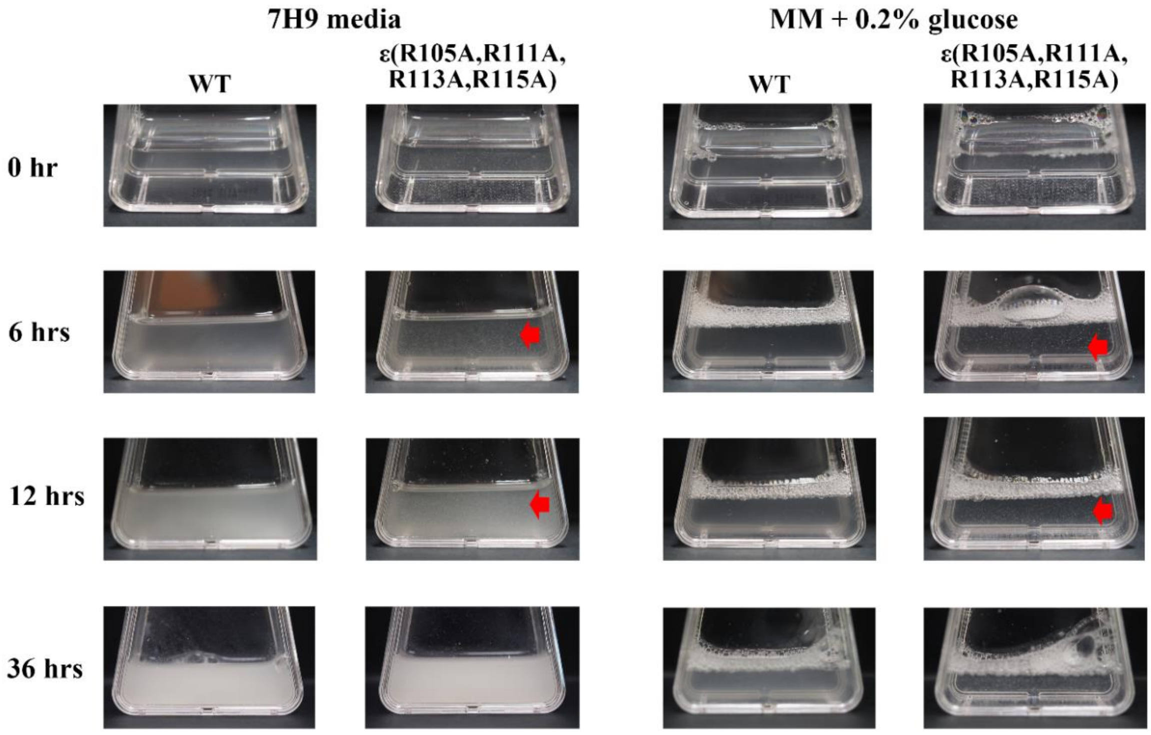

7H9 media
MM + 0.2% glucose
WT
ε(R105A,R111A, R113A,R115A)
WT
ε(R105A,R111A, R113A,R115A)
0 hr
6 hrs
12 hrs
36 hrs

(c)

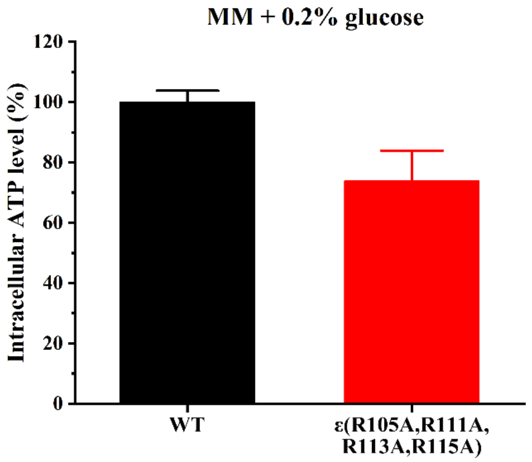

Supplementary Figure S1. Growth in liquid medium. (a) The optical density at wavelength of 600 nm of cultures of *M. smegmatis* WT (*black*), ε^{4A} mutant strain (*red*) and its complemented mutant strain (*blue*) in minimal media with 0.2% glucose after growing for 72 hrs. (b) Images of the *M. smegmatis* WT and ε^{4A} culture grown in 7H9 media and minimal media supplemented with 0.2% glucose at 0, 6, 12 and 36 hrs. The cell clumps (*red arrows*) were observed only in the ε^{4A} culture. (c) Intracellular ATP levels measured in WT and ε^{4A} mutant after growing for 72 hrs.

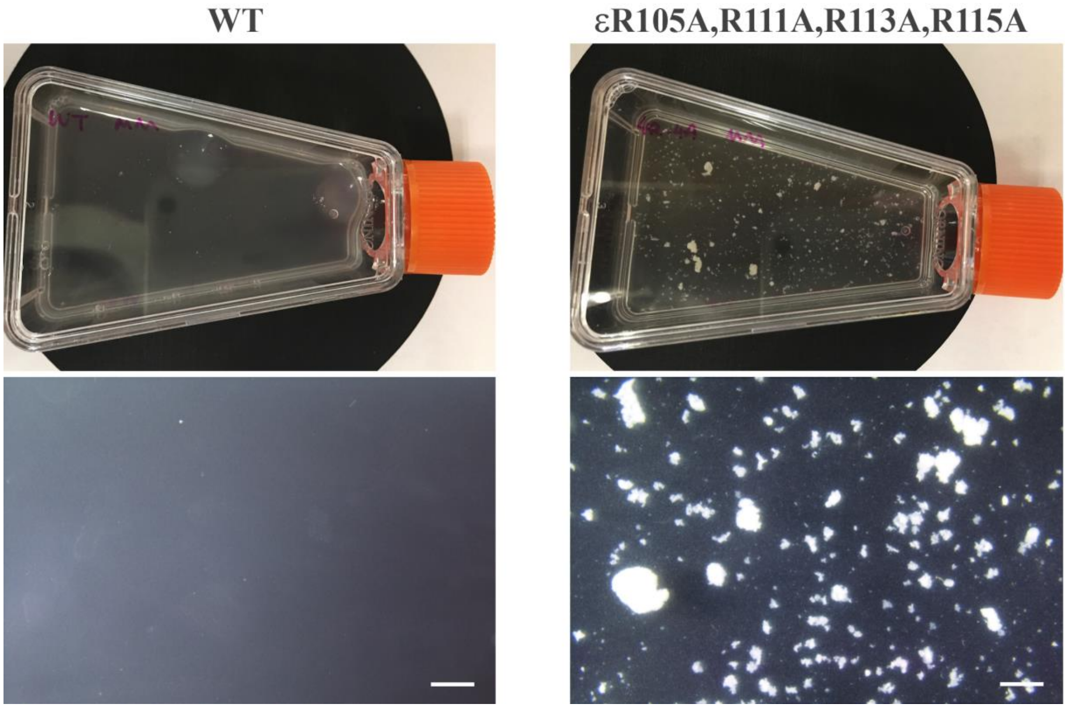

Supplementary Figure S2. Images of the *M. smegmatis* WT and ε^{4A} culture grown in minimal media. The mutant ε^{4A} culture forms clumps in the minimal media. White scale bar = 2 mm.

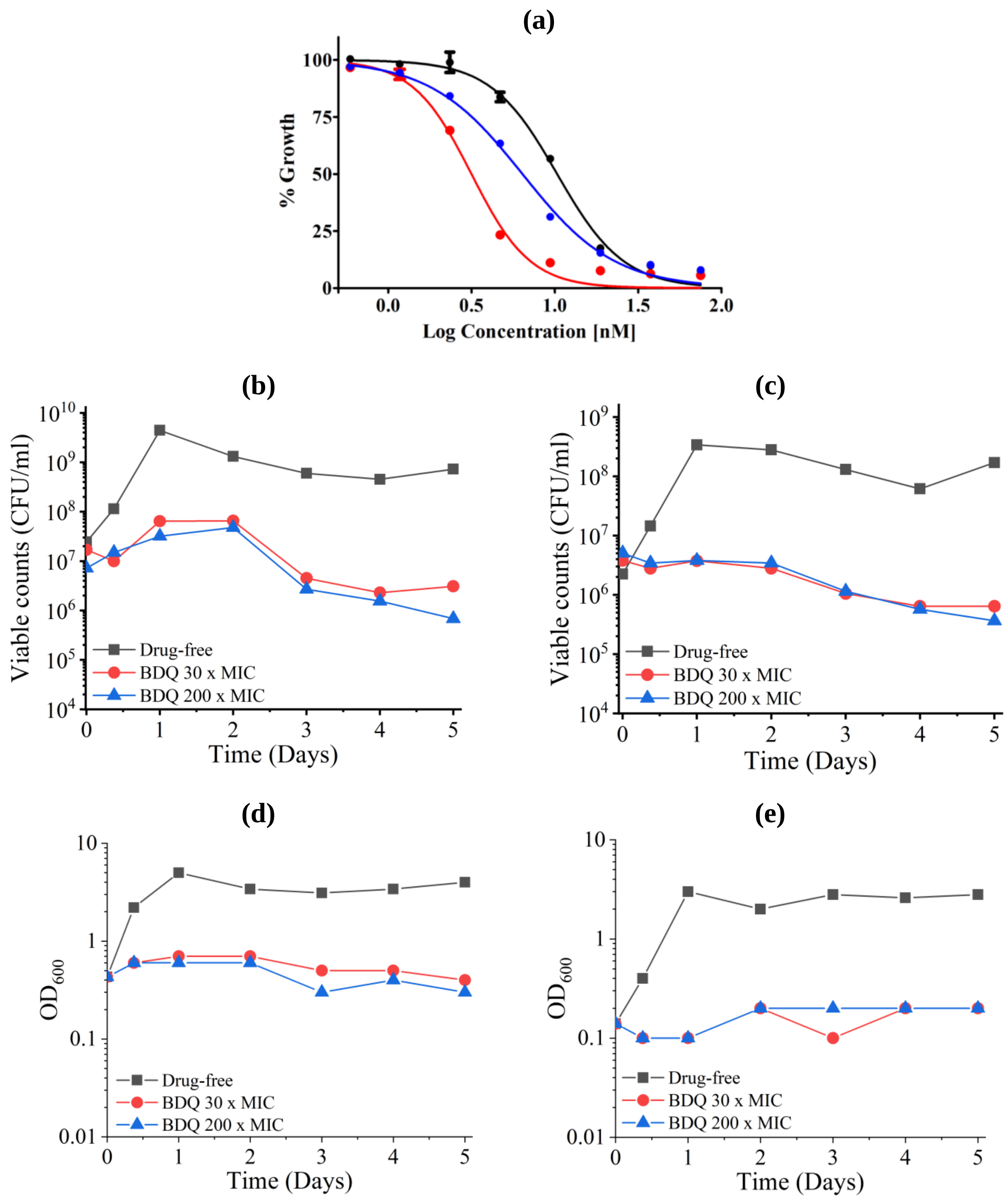

Supplementary Figure S3. Bedaquiline bacteriostatic and bacteriocidal effect on WT and mutant strains. (a) BDQ growth inhibition dose-response curves. *M. smegmatis* WT (*black*), mutant ε^{4A} (*red*) and the ε^{4A} complemented strain (*blue*). The experiments were performed in triplicate. Data are shown with their standard deviations. (b-e) Initial five days of BDQ kill kinetics against *M. smegmatis* mc^2 155 (b) WT or (c) the mutant ε^{4A} strain, and growth of (d) WT and (e) mutant strain in the presence of the drug. The bacteria were grown in liquid culture (LBT) in the presence of the indicated concentrations of BDQ. The experiments were repeated twice, and the profiles were identical.

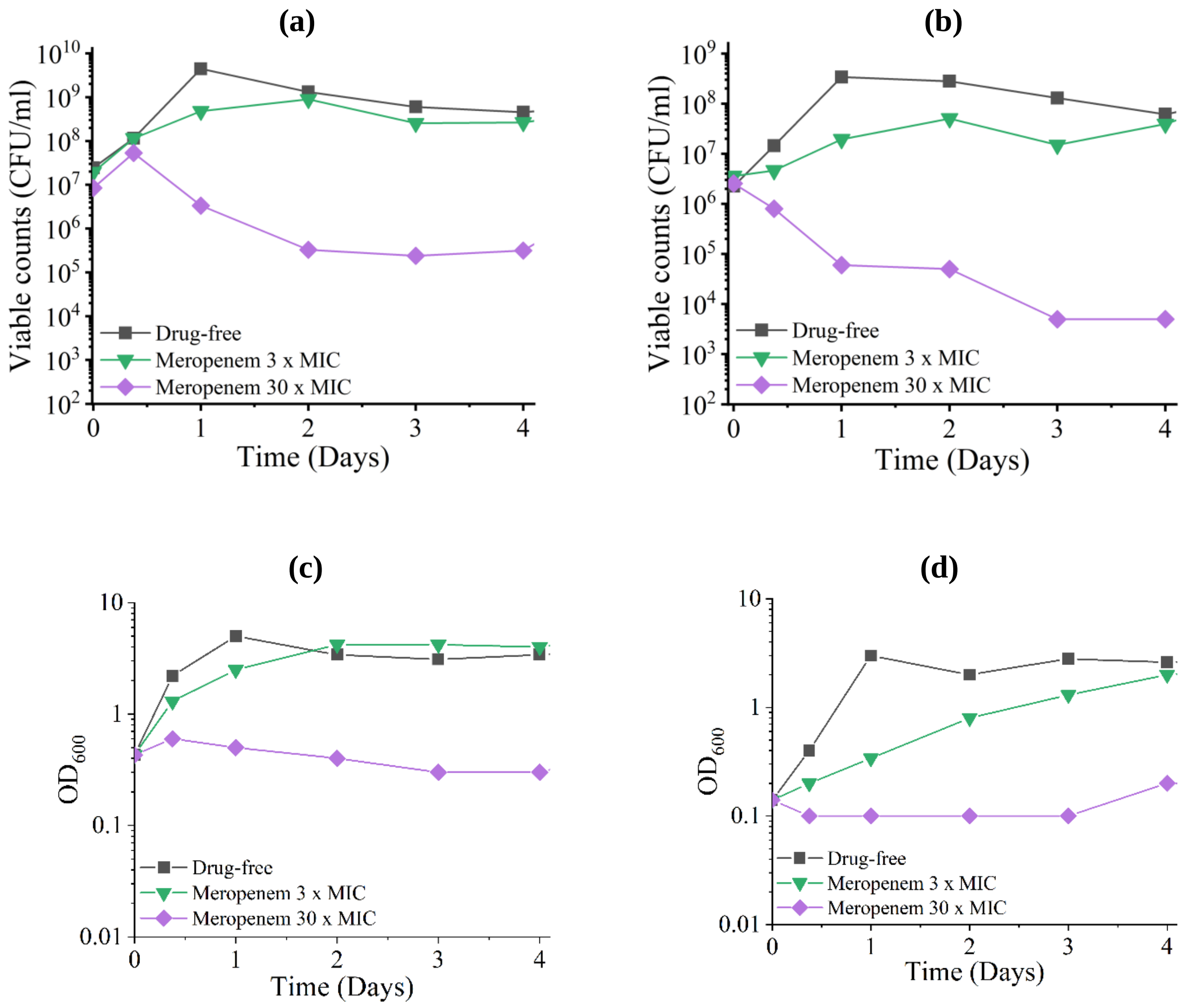

Supplementary Figure S4. Meropenem bacteriocidal effect on WT and mutant strains. (a-d) Initial four days of meropenem kill kinetics against *M. smegmatis* mc^2 155 (a) WT or (b) the mutant ε^{4A} strain. Growth of (c) WT and (d) mutant strain in the presence of the drug was observed at a 600 nm. The bacteria were grown in liquid culture (LBT) in the presence of the indicated concentrations of meropenem.

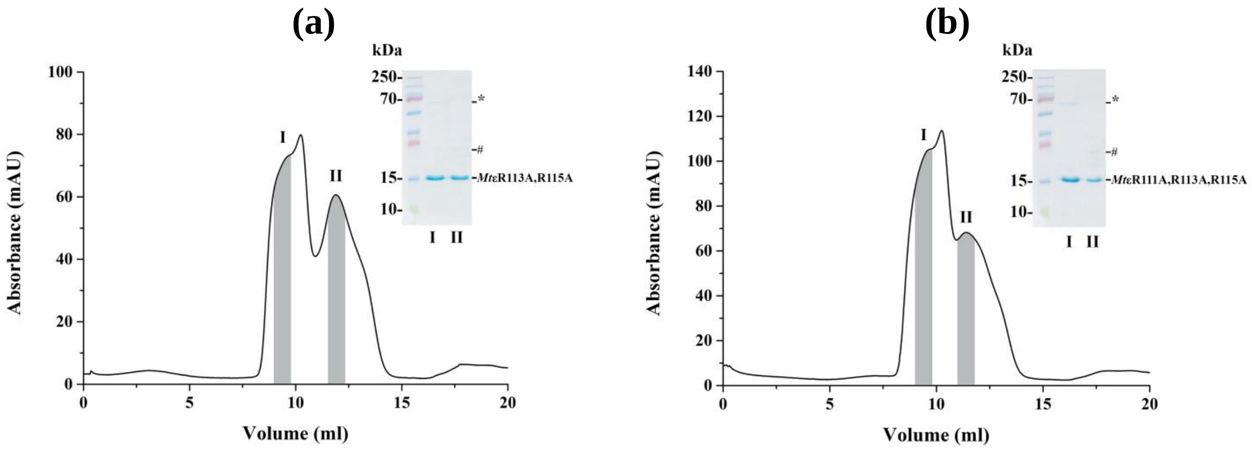

Supplementary Figure S5. SEC chromatogram and SDS gel of the *Mt*ε mutants (a) ε^{4A} and (b) ε^{4A}. Two peaks (I, II) were detected at an elution volume of around 9.5 and 11.5 ml. Grey area under each peak outlines the range of eluates collected. * and # indicate a higher- and lower oligomer, respectively.

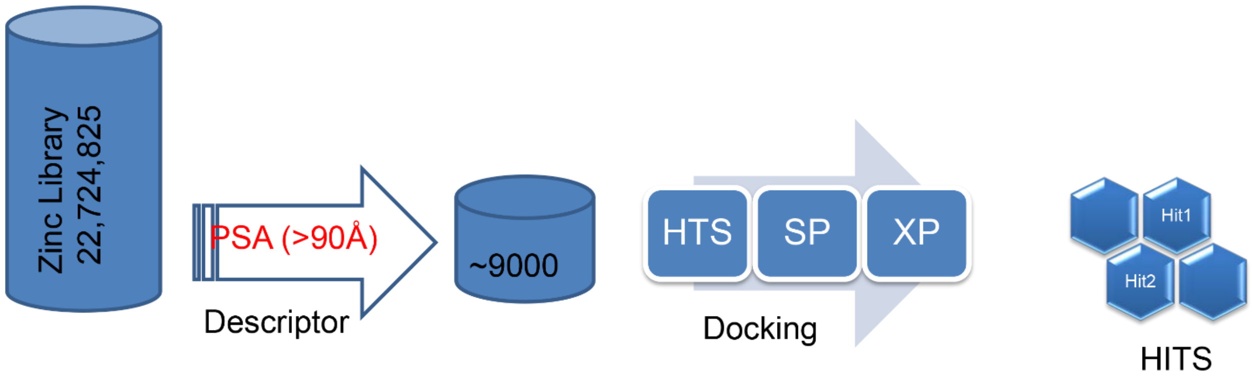

Supplementary Figure S6. Flowchart of virtual screening steps utilized in this study. The ligands from Zinc library were firstly screened based on the property filter, followed by glide docking based HTS, SP and XP score filters that ultimately led us to select 19 molecules for experimental studies.

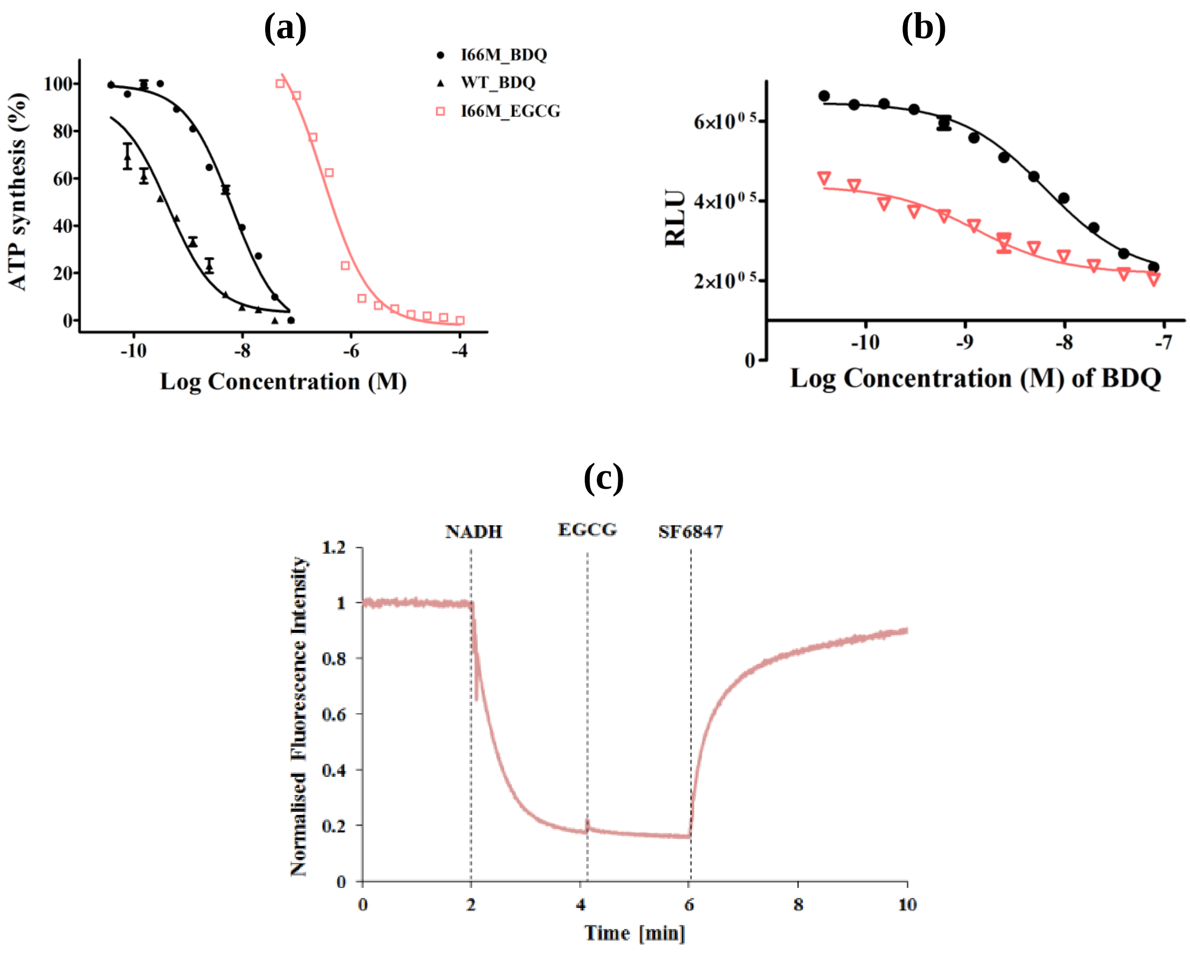

Supplementary Figure S7. Inhibition of ATP synthesis by BDQ (*black*) and EGCG (*rose*) on *M. smegmatis* I66M *c* subunit mutant IMVs with NADH succinate as substrate. (a) The *M. smegmatis* I66M mutant IMVs (●) reveal a 10-fold sensitivity compared to WT (▲), while the inhibitory profile of EGCG on *M. smegmatis* I66M mutant IMVs (□) did not alter. (b) Addition of 750 nM (△) of EGCG to increasing BDQ concentrations displays additional ATP synthesis inhibition of IMVs of the resistant *M. smegmatis* I66M *c* subunit mutant. (c) Fluorescence quenching of ACMA by WT IMVs after addition of 2 mM NADH. EGCG was added at a concentration of 200 nM at t = 3.5 min. The uncoupler SF6847 was added at the indicated time point to collapse the H^+-gradient. The experiments were performed in triplicate.

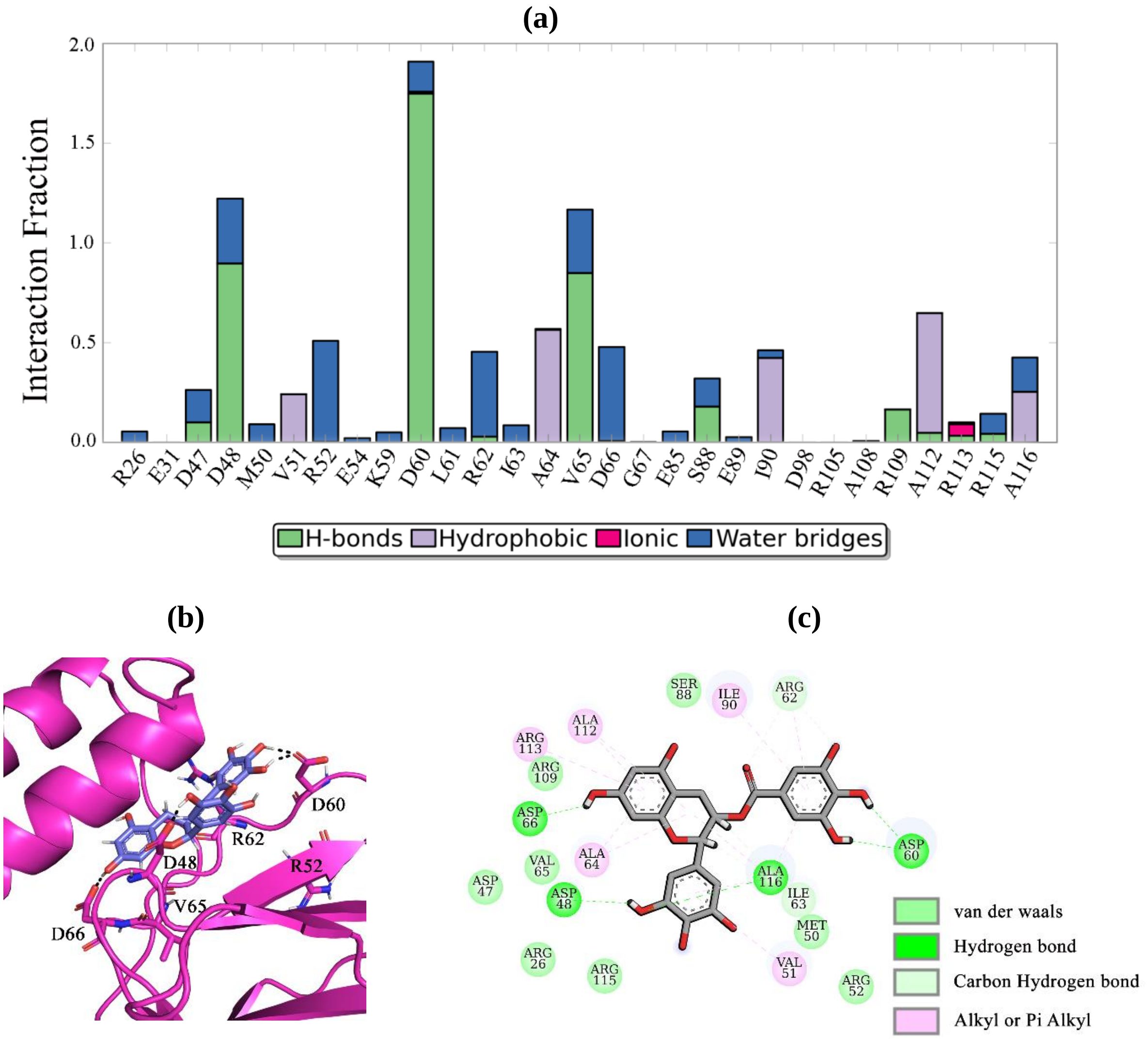

Supplementary Figure S8. Computational studies of interactions between *Mt*ε and EGCG. (a) Bar plot showing the stability of interactions of residues D48, D60, and V65 over 200 nanoseconds in fraction. (b) Cartoon model of binding pose of EGCG based on NMR titration data. Mainly, 7-OH group on the chroman ring mediates hydrogen bonding interaction with D66. The 3-OH- and 5-OH groups on 2-phenyl substitution on the chroman ring are involved in hydrogen bonding interactions with D48, A112 and A116. 3', 4'-hydroxyl groups on gallic acid ester were involved in hydrogen bond network with D60. Hydrophobic interactions of the three rings of EGCG with I90, A64, and A112 further stabilize its binding into the central cavity at the interface of N-terminal body and C-terminal α-helices of *Mt*ε. (c) 2D interaction map of EGCG on *Mt*ε highlights the important hydrogen bonding interactions in *green* and aromatic or aliphatic hydrophobic interactions in *pink*.

Table S1. Details of mutation(s) in ε^{4A} mutant strain based on whole genome sequencing

	Nucleotide position based on reference genome	***Mutated nucleotide(s)***	***Genes with mutation(s): Position of mutated amino acid***	***Remarks***
WT	N. A.	N. A.	N. A.	
ε^{4A} ***mutant***	304005	G > T	MSMEG_0269: D33E	- Protein name: 3-oxoacyl-ACP reductase<br>- Involved in fatty acid biosynthesis[26]<br>- Conservative mutation
	3998526	G > A	MSMEG_3929: D15E	- Protein name: [NiFe] hydrogenase subunit delta<br>- Involved in conversion of NAD(P)H to hydrogen under hypoxia[27]<br>- Conservative mutation
	4140012	GCAT > G	MSMEG_4066: ΔM425	- Protein name: Dipeptidyl aminopeptidase/acylaminoacyl peptidase<br>- Catalysed aminopeptidase activity[28]<br>- Single amino acid deletion
	5032246<br>5032247<br>5032248	G > C<br>C > G<br>G > C	*atpC*: R115A	- Protein name: F-ATP synthase subunit epsilon<br>- Catalysed ATP synthesis coupled by proton transport[28]<br>- Non-conservative mutations
	5032253<br>5032254	C > G<br>G > C	*atpC*: R113A	
	5032258<br>5032259<br>5032260	C > G<br>C > G<br>G > C	*atpC*: R111A	
	5032277<br>5032278	C > G<br>G > C	*atpC*: R105A	

Three additional mutations were found on the mutant's genome as described in detail in the Supplementary Table 1 above. Two mutations were conservative mutations, where the residue Asp was replaced by a Glu and were identified in the 3-oxoacyl-ACP reductase and [NiFe] hydrogenase subunit delta, respectively. In case of the 3-oxacyl-ACP reductase an atomic resolution structure is available (PDB ID: 6CI8)[29]. A careful look at this structure displayed that the mutated residue is on the protein surface, and its side chain is pointed outwards and does not have any non-covalent interaction with other amino acids. Therefore, this conservative mutation is unlikely to change the protein structure and function or to have any effect on catalysis. The third mutation is a single amino acid deletion on the gene encoded for aminopeptidase activity, which is not involved in the oxidative phosphorylation pathway or cell wall synthesis.

Table S2. Details of the primers and templates used in each PCR, as well as the product obtained

PCR		Description
**I**	Forward primer	5’-CGC CGC ATT GCG CGC CGT CG-3’
	Reverse primer	5’-GCA ATG CGG CGC GGC CCC TGG-3’
	Template	pET9d-*Mtb*AtpC[19]
	Product	pET9d-*Mtb*AtpCR113A
**II**	Forward primer	5’-CAT TGG CAG CCG TCG GCG CGA TC-3’
	Reverse primer	5’-CGG CTG CCA ATG CGG CGC GG-3’
	Template	pET9d-*Mtb*AtpCR113A
	Product	pET9d-*Mtb*AtpCR113A, R115A
**III**	Forward primer	5’-GGG CGC CGC CGC ATT GGC AGC-3’
	Reverse primer	5’-GCG GCG CCC CTG GCA GCG ATG-3’
	Template	pET9d-*Mtb*AtpCR113A, R115A
	Product	pET9d-*Mtb*AtpCR111A, R113A, R115A
**IV**	Forward primer	5’-ATC CCG CCA TCG CTG CCA GGG-3’
	Reverse primer	5’-GCG ATG GCG GGA TCG TCG GAT TCG G-3’
	Template	pET9d-*Mtb*AtpCR111A, R113A, R115A
	Product	pET9d-*Mtb*AtpCR105A, R111A, R113A, R115A

[#]The mutated nucleotides were underlined.
